# Persistently high hepatitis C rates in haemodialysis patients in Brazil [a systematic review and meta-analysis]

**DOI:** 10.1038/s41598-021-03961-x

**Published:** 2022-01-10

**Authors:** Roberta Pereira Niquini, Jurema Corrêa da Mota, Leonardo Soares Bastos, Diego da Costa Moreira Barbosa, Juliane da Silva Falcão, Paloma Palmieri, Patrícia Martins, Livia Melo Villar, Francisco I. Bastos

**Affiliations:** 1 Federal Institute of Education, Science, and Technology of Rio de Janeiro (IFRJ), Rio de Janeiro, Brazil; 2 Institute of Scientific and Technological Communication and Information in Health, Oswaldo Cruz Foundation (ICICT-FIOCRUZ), Biblioteca de Manguinhos suite 229, Av. Brasil 4365, Rio de Janeiro, 21045-900 Brazil; 3 Program for Scientific Computing, Oswaldo Cruz Foundation (PROCC-FIOCRUZ), Rio de Janeiro, Brazil; 4 Laboratory of Viral Hepatitis, Oswaldo Cruz Institute, Oswaldo Cruz Foundation (IOC-FIOCRUZ), Rio de Janeiro, Brazil

**Keywords:** Biomarkers, Gastroenterology, Health care, Nephrology, Risk factors

## Abstract

We conducted a systematic review and meta-analysis of studies assessing HCV infection rates in haemodialysis patients in Brazil (Prospero CRD #42021275068). We included studies on patients under haemodialysis, comprising both convenience samples and exhaustive information from selected services. Patients underwent HCV serological testing with or without confirmation by HCV RNA PCR. Exclusion criteria were the following: absence of primary empirical information and studies without information on their respective settings, study year, accurate infection rates, or full specification of diagnostic tests. Studies with samples ≤ 30 and serial assessments with repeated information were also excluded. Reference databases included PubMed, LILACS, Scopus, and Web of Science for the period 1989–2019. A systematic review was carried out, followed by two independent meta-analyses: (i) studies with data on HCV prevalence and (ii) studies with a confirmatory PCR (i.e., active infection), respectively. A comprehensive set of different methods and procedures were used: forest plots and respective statistics, polynomial regression, meta-regression, subgroup influence, quality assessment, and trim-and-fill analysis. 29 studies and 11,290 individuals were assessed. The average time patients were in haemodialysis varied from 23.5 to 56.3 months. Prevalence of HCV infection was highly heterogeneous, with a pronounced decrease from 1992 to 2001, followed by a plateau and a slight decrease in recent years. The summary measure for HCV prevalence was 34% (95% CI 26–43%) for studies implemented before 2001. For studies implemented after 2001, the corresponding summary measure was 11% (95% CI 8–15%). Estimates for prevalence of active HCV infection were also highly heterogeneous. There was a marked decline from 1996 to 2001, followed by a plateau and a slight increase after 2010. The summary measure for active HCV infection was 19% (95% CI 15–25%) in studies carried out before 2001. For studies implemented after 2001, the corresponding summary measure was 9% (95% CI 6–13%). Heterogeneity was pervasive, but different analyses helped to identify its underlying sources. Besides the year each study was conducted, the findings differed markedly between geographic regions and were heavily influenced by the size of the studies and publication biases. Our systematic review and meta-analysis documented a substantial decline in HCV prevalence among Brazilian haemodialysis patients from 1992 to 2015. CKD should be targeted with specific interventions to prevent HCV infection, and if prevention fails, prompt diagnosis and treatment. Although the goal of HCV elimination by 2030 in Brazil remains elusive, it is necessary to adopt measures to achieve micro-elimination and to launch initiatives towards targeted interventions to curb the spread of HCV in people with CKD, among other high-risk groups. This is of particular concern in the context of a protracted COVID-19 pandemic and a major economic and political crisis.

## Introduction

Hepatitis C infection is widespread in the world, despite concerted efforts by international agencies such as the World Health Organization (WHO) and different national health systems to curb the disease and ideally to eliminate it by 2030^1^. Currently, the prospects for timely elimination of hepatitis C remain elusive, and several authors have focused on the concept of micro-elimination, i.e., elimination of hepatitis C in some populations and contexts^2^. Unfortunately, this is not the case of people with CKD in haemodialysis. They continue to be disproportionately affected by hepatitis C and several other infections^3^.

Severe renal failure (SRF) is a serious, potentially life-threatening medical condition. SRF is a likely outcome of chronic kidney disease (CKD) when several underlying conditions such as diabetes, hypertension, chronic renal infection, and renal cancer may lead to a progressive decrease in the glomerular filtration rate (GFR) and the decrease cannot be reversed or at least substantially ameliorated^4^. SRF can evolve to end stage kidney disease.

The management of end stage kidney disease usually includes haemodialysis and kidney transplantation. Dialysis is key, since it is both an essential strategy for keeping patients with severe renal failure alive and healthy as well as an interim procedure to keep patients fit for kidney transplantation^5^. In Brazil, the process mediating the demand for kidney transplantation has been both complex and slow^6^.

Patients with SRF have to deal with a severe medical condition, subjected to a chronic, invasive intervention, namely haemodialysis, and suffering from the stress and anxiety associated with long waiting lines for transplantation^7^ (on waiting lines for lung transplantation and associated psychological distress).

Despite the efficient control of HCV infection in this group of patients in some high-income countries, this is unfortunately not the case of Brazil, a middle-income country with a population of more than 213 million. Considering both the persistently high HCV infection rates over the years as well as the magnitude of even relatively rare conditions when considering the high numbers of patients, it is crucial to address this challenge in Brazil.

The current article focuses on one such infection or disease: HCV infections or hepatitis C among haemodialysis patients. We conducted a systematic review and meta-analysis of studies assessing HCV infection rates in haemodialysis patients in Brazil.

## Background

We conducted a systematic review and meta-analysis of studies assessing HCV infection rates in haemodialysis patients in Brazil (Prospero CRD #42021275068).

The main objective of the review and meta-analysis was to assess the HCV infection rates and prevalence of active infection among patients under haemodialysis. This information is key to any effort to achieve micro-elimination of hepatitis C^8^ and to call the attention of policymakers, administrators, and health professionals to the pressing need to prevent HCV infection in this population and to provide comprehensive care and prompt treatment when infection takes place.

In full agreement with PROSPERO’s registration rules and the PRISMA statement (see Web Appendix 6), we conducted a comprehensive search and extraction of references as detailed in the Web Appendix 1. This search and extraction included papers from 2020, documenting a dire picture of the SARS-CoV-2 pandemic in Brazil and of services affected by dramatic budgetary constraints. Although these articles were not included in the present study, since major contextual changes and competitive risks of dying from COVID-19 confound the findings from previous studies, they lent a new purpose to the current study: to function as a baseline for a future when the COVID-19 epidemic is curbed in Brazil and the various levels of health services are fully restored^9^.

## Methods

The systematic search deployed a procedure conducted in the reference databases as follows: PubMed, Scopus, Web of Science/ISI (Core Collection), and LILACS. The search covered articles published up to December 31, 2019. The procedures and scripts used in the search process are available in Web Appendix 1.

Programming uses the tools provided by the databases themselves. Data were extracted using Zotero^10^. A preliminary search and extraction were performed by three authors of the current article (RPN, DCMB, and JSF), using a blinded procedure and the search terms defined in the article´s original version.

In agreement with the requirements by PROSPERO and the suggestions submitted to the authors by an anonymous reviewer, search strategies were redefined and substantially broadened. Entirely new searches were performed, as well as a new round of extraction. This second and final procedures were performed independently by two other authors (JCM and FIB). The second search and extraction allowed the authors to double-check previous findings and to implement a comprehensive quality assessment of the articles.

Papers from 2020 were searched and extracted, but not included in our study. The COVID-19 epidemic has heavily affected the Brazilian healthcare system as a whole and specifically hit the haemodialysis centres. The latter are on the verge of collapse. The full impact of the COVID-19 epidemic on haemodialysis has still not been properly evaluated, and information remains far from comprehensive^11^.

Additional searches were performed in LILACS using the equivalent terms in Spanish and Portuguese.

### Inclusion criteria

Articles included in this study followed three basic criteria:

1. Assessment of the target population (haemodialysis patients in Brazilian healthcare units) whether or not as the respective article’s exclusive focus;
2. Probabilistic samples of the population covered by a given healthcare unit or network of facilities, or adoption of a census approach (i.e., aimed to include all patients from a given setting/service or network of facilities);
3. Performance of HCV serological tests with or without further confirmation using HCV RNA PCR (polymerase chain reaction).

### Exclusion criteria

The following criteria were used to exclude articles from the current review:

1. Studies exclusively based on secondary data, with no first-hand empirical data (i.e., the researchers asked local managers to send the data, but did not double-check the data through any on-site assessment);
2. Failure to provide basic information on the sites where the studies were performed (at least the municipality or state of Brazil). There were no nationwide studies. A survey by the Brazilian Society of Nephrology was not a nationwide study but a compilation of data from responses summarized via surface mail, as discussed in detail below;
3. Lack of information on the year the study was performed. Multi-year studies comprising serial assessments of the same service were presented as individual studies per year, that is, as many times as the assessment took place. No longitudinal studies were identified;
4. Lack of information on the sample size, or samples with fewer than 30 patients (in order to avoid the small numbers fallacy^12^;
5. Lack of information on HCV infection rates in the target population or insufficient reliable data that might help calculate the rate;
6. Lack of information on the test(s) used to diagnose HCV infection;
7. Studies that used no additional tests besides first and second-generation ELISA/EIA (since their lower sensitivity and specificity can introduce further heterogeneity and less accuracy in the efforts to find reliable pooled estimates of point prevalence and respective confidence intervals)^13,14^;
8. Studies that only provided data on “dialysis”, without further specifying subsets of patients in peritoneal dialysis versus haemodialysis and the respective prevalence rates;
9. Studies in a series assessing the same sample. In this case, our review only included the most comprehensive study in the series (to avoid duplicate/multiple counts of the same group of patients).

The criteria are consistent with the CoCoPop guidelines, which are fully described in the Web Appendix 2. The review followed the standard steps and procedures in full agreement with the PRISMA statement and different tutorials (e.g. https://guides.library.cornell.edu/evidence-synthesis/service). Steps and procedures are fully described in Web Appendix 1.

### Steps and procedures

The first step was a detailed reading by independent reviewers of the titles of all articles selected with the search algorithm. Articles with no clear link to the study’s purpose and inclusion criteria were excluded before any further steps.

The second step consisted of the analysis of the contents of abstracts from all the articles approved in step 1. Abstracts were screened for their relevance to the study’s objectives and criteria. When the abstracts/articles were consistent with these criteria, the full texts were read by the reviewers.

The reviewers extracted core information from the articles selected for full-text reading, using a standard form completed independently by each of the three reviewers. The standard form included the following variables: name of the first author; the year(s) the study was implemented and concluded; the major geographic region of Brazil (among five) where the study was conducted; the target population/patient group; sample size; laboratory tests used for diagnosis of HCV infection; prevalence of HCV infection; proportion (%) of individuals who had ever received blood/blood products; average age (in years) of the haemodialysis patients; and average time on haemodialysis. Averages were defined as arithmetic means. In the absence of means, the medians or interpolated values were used.

The fourth and final step consisted of the application of the exclusion criteria to all the articles. When the three independent reviewers failed to reach the same decision, the article was discussed until a consensus was reached.

### Data analysis

Two independent meta-analyses of the data were carried out. The first included all studies with data on HCV prevalence, defined by third-generation ELISA (enzyme-linked immunosorbent assay) (hereinafter “III”), MEIA (microparticle enzyme immunoassay), or EIA (enzyme immunoassay).

Articles in which HCV prevalence was defined according to positive ELISA III results with or without additional tests such as LIA (line immunoassay) were included. For articles in which HCV prevalence was defined according to CLIA (chemiluminescence immune assay) and ECLIA (electro-chemiluminescence immunoassay) (taken together), this option was also defined for our purposes as a valid diagnosis of HCV infection.

The second analysis included all studies selected for the first analysis with a confirmatory test using PCR (polymerase chain reaction). The combination indicates active infection, i.e., that HCV persists in the individual and viral load exceeds the detection threshold.

The findings from the two analyses were summarized and displayed as graphs showing HCV prevalence according to the year the study was implemented (to distinguish between the time when the fieldwork began and the respective study’s actual publication). The five major geographic regions of Brazil were displayed with different colours. The major geographic regions represent the gross geographic division of the country, as defined by the Brazilian Institute of Geography and Statistics (IBGE).

The analyses and corresponding graphs made the following assumptions: (i) the number of HCV-positive patients in each study *Y* followed a binomial distribution, with a parameter *n* corresponding to the number of patients tested in the context of each study and (ii) the a priori distribution was also assumed as uniform (0, 1) for the different studies.

The subsequent distribution of studies was defined as a beta (β) distribution (y + 1, n-y + 1). The 95% credibility intervals were calculated based on this distribution.

The graphs were produced by fitting a local polynomial regression (*loess*), taking the year each study was launched and a posteriori mean prevalence as the predictor.

Based on data and graphs, study heterogeneity (I^2^ statistics) was calculated^15^. Study heterogeneity was classified according to the criteria proposed by Higgins et al.^16^: absence of any relevant heterogeneity for I^2^ values close to 0%; and heterogeneities defined as low, moderate, and high for levels of 25%, 50%, and 75%, respectively.

Meta-analyses were implemented to calculate the summary statistics, comprising point estimates and their respective 95% confidence intervals (95% CIs). The studies were divided into two subsets, before versus after 2001. This year was defined as the cut-off, because after 2001 standards for the operation of health units performing haemodialysis were issued by the Brazilian Ministry of Health (BMoH)^17,18^. We also estimated prevalence rates and respective 95% CIs for each major geographic region of Brazil, before and after 2001, using random effects models.

All the analyses were performed with the open-source software R 3.6.3^19^. The R scripts are available on request.

A comprehensive set of complementary analyses based on classic statistics was carried out and is available in Web Appendix 3.

The statistics comprise the analyses as follows, in full compliance with the PRISMA statement and respective checklist, as available in the specific final Web Appendix 6: forest plots and their respective statistics, subgroup analysis, meta-regression, and influence analysis, as well as the assessment of publication biases using the trim-and-fill statistics based on funnel plot graphs.

The findings are briefly mentioned in the body of the article and are presented and discussed in detail in the abovementioned Web Appendix 3.

The quality assessment is summarized in Web Appendix 4. After the extraction of articles from 2020, which were not included in this study, we realized that information on tests might be confused by suboptimal descriptions. We decided to double-check all information vis-à-vis a comprehensive spreadsheet downloaded from the ANVISA website and further verified after a formal request to the agency (Protocol 2021195961). Full information is available in Web Appendix 5.

## Results

The final search strategy yielded 292 articles (54 from PubMed/Medline, 76 from SCOPUS, 58 from Web of Science, and 87 from LILACS). Due to overlapping, 58 duplicate articles (retrieved from more than one database) were excluded.

**Figure 1:**
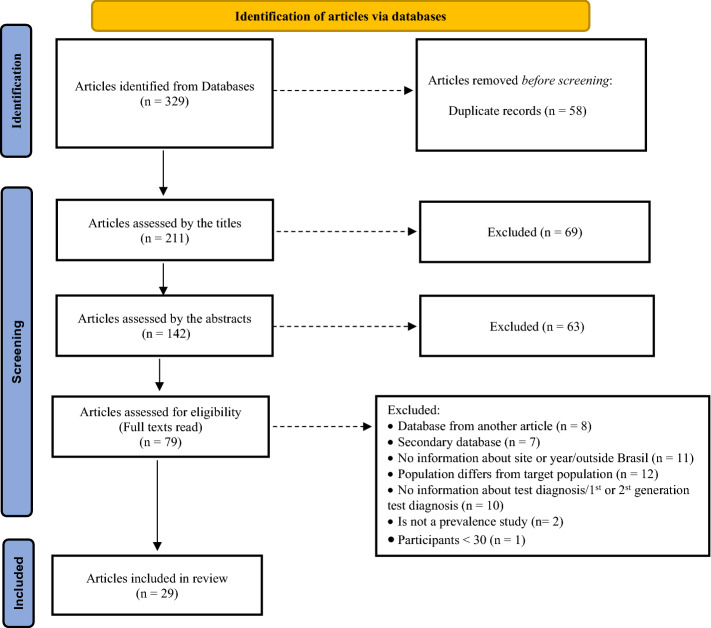
Flow of information and stages of the systematic review.

Figure 1 shows the results of the successive steps and their respective numbers.

Among the remaining 211 articles, 69 were excluded after independent assessments of their titles. Sixty-three were excluded after assessing their abstracts. Seventy-nine articles were then read as full texts. After this last step, 29 articles were selected for the systematic review and meta-analysis. The new round of searches and extraction after registration in PROSPERO is described in full in Web Appendix 1 and was included in the revised Fig. 1.

Two studies^20,21^ presented data for three periods (1993, 1996, and 1999) and two periods (2000–2002 and 2006), respectively. Data were stratified according to the above-mentioned periods of data collection. Thus, although 29 articles were analysed, the N for point prevalence and respective 95% CIs was 32 (Table 1).

**Table 1:** Prevalence rates of HCV infection in haemodialysis patients in Brazil according to major geographic region in which the study was conducted, year of start of study, and study population/sample size.

Author (year)	Region of Brazil	Year of start of study	Year of end of study	n	Hepatitis C prevalence (prior contact with HCV)	Hepatitis C prevalence (active HCV infection)
de Oliveira et al., 2001^22^	SE	1992	1992	125	68.00	–
Carneiro et al., 2005^20^	CO	1993	1993	153	28.20^a^	–
Carneiro et al., 2005^20^	CO	1996	1996	282	34.70^a^	–
Carrilho et al., 2004^26^	S	1996	1997	813	33.95	21.03
de Medeiros et al., 2004^27^	NE	1997	1997	746	52.28	–
Moreira et al., 2003^28^	SE	1997	1998	281	11.74	8.19
Carneiro et al., 2001^29^	CO	1998	1998	428	43.22	24.77^f^
Carvalho et al., 1999^24^	S	1998	1998	74	39.19	27.03
Carneiro et al., 2005^20^	CO	1999	1999	451	37.80^a^	–
Busek et al., 2002^30^	SE	2000	2000	265	23.77^b^	22.64
Callegaro et al., 2006^21^	S	2000	2002	71	21.13	16.90
Dotta et al., ^31^	S	2000	2000	128	31.25	18.75
Choi et al., 2003^32^	S	2001	2001	47	36.17	–
Souza et al., 2003^33^	N	2001	2001	100	13.00	11.00
Carneiro et al., 2007^34^	CO	2002	2002	1095	16.44	10.96
de Albuquerque et al., 2005^35^	NE	2002	2002	250	8.40	7.60
Florentino, 2004^36^	NE	2002	2002	188	4.79^c^	3.70
Santos and Souto, 2007^37^	CO	2002	2005	433	16.86^d^	–
Silva et al., 2006^38^	NE	2002	2002	1243	10.46	7.40
Bessa et al., 2009^39^	NE	2003	2003	241	9.13	–
Freitas et al., 2008^40^	CO	2003	2003	163	11.66	7.36^f^
Galperim et al., 2010^41^	S	2005	2006	325	32.92	20.92
Callegaro et al., 2006^21^	S	2006	2006	70	10.00	2.86
Maia et al., 2009^42^	N	2006	2007	395	13.92	–
Leão et al., 2010^43^	SE	2007	2007	236	14.83	10.59
da Silva et al., 2013^44^	S	2009	2010	159	23.27	23.27
Fontenele et al., 2015^45^	NE	2010	2013	301	4.98	4.65
Rodrigues de Freitas et al., 2013^46^	N	2011	2011	798	8.40	5.26
Santos, et al., 2017^47^	NE	2011	2011	605	3.14^e^	–
Vidales-Braz et al., 2015^48^	S	2012	2013	287	19.16	19.16
Barbosa JR et al., 2017^49^	NE	2014	2015	143	12.59	–
Cordeiro VM et al., 2018^23^	N	2014	2015	394	2.80	–

Studios that used different diagnostic method: ^a^ELISA + and LIA + ; ^b^EIA III + ; ^c^ELISA III + or Detect^®^ for HCV, version 3.0, ALKA laboratory; ^d^MEIA III + ; ^e^CLIA + and ECLIA + ; ^f^LIA + and PCR +.

Overall, 28.1% (n = 9) studies were based on data from the South of Brazil, 25.0% (n = 8) from the Northeast, 21.9% (n = 7) from the Central-West, and 12.5% (n = 4) from both the North and the Southeast. A total of 11,290 individuals were assessed in the pooled analyses, 32.9% (n = 3717) of which from the Northeast, 26.6% (n = 3005) from the Central-West, 17.5% (n = 1974) from the South, 15.0% (n = 1687) from the North, and 8.0% (n = 907) from Southeast (Table 1). More than half (62.5% [n = 20]) of the studies were conducted after 2001 (Table 1).

The point prevalence estimates varied substantially, from as high as 68.0% in a study in 1992 in Southeast Brazil^22^ to as low as 2.8% in a study in 2014 in the North^23^. Studies addressing active HCV infection, i.e., consisting of patients that tested for HCV antibodies and HCV-RNA, also varied considerably, but not as much as in the previous studies. The highest point prevalence for active HCV infection was 27.3%, in a study in 1998 in the South^24^, whilst the lowest was 2.9%, in a study in 2006, also in the South^21^ (Table 1).

Besides the comprehensive list presented in Table 1, findings were depicted as forest plots (Web Appendix 3). Summary measures and respective statistics using both fixed effects models (FEM) and random effects models (REM) are presented in Web Appendix 3.

Findings from all the analyses are presented, despite some occasional minor discrepancies may be observed. We opted for a full presentation of all the findings, aiming to improve transparency and reproducibility^25^.

Considering the above-mentioned stratification of two articles (n = 32 for 29 articles), 34.4% (n = 11) were conducted in a single healthcare unit where haemodialysis was performed, whilst 15.6% (n = 5) analysed data from a pool of 10 or more facilities. The great majority of articles (96.9%), considering the same stratification, followed a census design, i.e., researchers ordered testing for each single patient. However, 62.5% (n = 20) did not provide any information on refusals (Table 2 and Web Appendix 2). Since refusals and non-response are likely to be differential, the absence of this information represents a limitation.

**Table 2:** Characteristics of study populations in articles selected to estimate prevalence rates of HCV infection in haemodialysis patients in Brazil.

Author (year)	Population or sample (% refusals)	Age (in years)			Duration of haemodialysis in months (central tendency)	Lifetime blood transfusion (%)
		Central tendency	Min	Max		
de Oliveira et al., 2001^22^	Pop. from 1 clinic (NI)	–	–	–	–	–
Carneiro et al., 2005^20^	Pop. from 8 clinics (NI)	42.2	–	–	32.9^a^	–
Carneiro et al., 2005^20^	Pop. from 10 clinics (NI)	43.3	–	–	28.0^a^	–
Carrilho et al., 2004^26^	Pop. from 22 clinics (0%)	47.1	14	86	32.1	64.9
de Medeiros et al., 2004^27^	Pop. from 12 clinics (0%)	43.5	10	86	–	90.7
Moreira et al., 2003^28^	Pop de 2 clinics (NI)	–	18	–	–	–
Carneiro et al., 2001^29^	Pop. from 8 clinics (0%)	46.1	9	70	–	–
Carvalho et al., 1999^24^	Pop. from 1 clinic (NI)	49.8	–	–	41.4	–
Carneiro et al., 2005^20^	Pop. from 8 clinics (NI)	45.3	–	–	40.8^a^	–
Busek et al., 2002^30^	Pop. from 2 clinics (NI)	–	–	–	–	–
Callegaro et al., 2006^21^	Pop. from 1 clinic (NI)	–	–	–	–	–
Dotta et al., ^31^	Pop. from 3 clinics (NI)	56.5	–	–	48.6	52.3
Choi et al., 2003^32^	Pop. from 1 clinic (NI)	50.3	–	–	49.6	–
Souza et al., 2003^33^	Pop. from 1 clinic (0%)	47.6	13	82	23.5^a^	96.0
Carneiro et al., 2007^34^	Pop de 15 clinics (0%)	49.3	3	97	30.8^a^	82.2
de Albuquerque et al., 2005^35^	Pop. from 1 clinic (3%)	46.0	17	92	52.3^a^	64.4
Florentino, 2004^36^	Pop. from 4 clinics (NI)	46.2	12	84	29.0	44.1
Santos and Souto, 2007^37^	Pop. from 6 clinics (NI)	50.0	–	–	43.0	84.1
Silva et al., 2006^38^	Pop. from 10 clinics (NI)	–	–	–	–	–
Bessa et al., 2009^39^	Pop. from 1 clinic (0%)	–	–	–	–	–
Freitas et al., 2008^40^	Sample, 5 clinics (NI)	48.0	13	83	40.9^a^	80.4
Galperim et al., 2010^41^	Pop. from 4 clinics (NI)	54.4	22	90	56.3	76.0
Callegaro et al., 2006^21^	Pop. from 1 clinic (NI)	–	–	–	–	–
Maia et al., 2009^42^	Pop de 1 clinic (NI)	–	–	–	–	–
Leão et al., 2010^43^	Pop. from 1 clinic (0%)	55.1	–	–	46.2	74.0
da Silva et al., 2013^44^	Pop. from 2 clinics (NI)	56.9	18	–	55.1	94.3
Fontenele et al., 2015^45^	Pop. from 2 clinic (12%)	49.0	11	84	40.0	32.9
Rodrigues de Freitas et al., 2013^46^	Pop de 7 clinics (1%)	49.0	18	88	40.7^a^	–
Santos, et al., 2017^47^	Pop. from 4 clinics (5%)	47.9^b^	18	–	47.1^b^	–
Vidales-Braz et al., 2015^48^	Pop. from 3 clinics (NI)	–	–	–	–	–
Barbosa et al., 2017^49^	Pop. from 1 clinic (NI)	48.0	18	–	–	62.9
Cordeiro et al., 2018^23^	Pop. from 4 clinics (7%)	53.4	18	90	38.7^a^	76.3

*NI* not informed, ^a^median calculated via linear interpolation of accumulated distribution of time in dialysis; ^b^weighted mean of medians.

Considering the 23 studies with data on the central tendency measures of patients’ age, the lowest age was 42.2 years^20^ and the highest was 56.9 years^44^. The weighted average for the 23 studies with available data on age was 48.3 years, with lower ages in studies before 2001 (n = 8; 45.7 years) and higher ages (49.9 years) in studies after 2001 (n = 15) (Table 2).

Information on age range was available in 16 studies. Half of the studies only included patients over Brazil’s age of majority (18), whilst the other half also included children and adolescents. Data on the latter (numbers and/or proportions) were not available in the original studies and could not be obtained from the authors (Table 2 and Web Appendix 2).

The average time patients were in haemodialysis varied from 23.5 months^33^ to 56.3 months^41^. The weighted average (considering studies with available data [n = 20] and their respective samples) was 39.3 months; lower (35.1 months) for studies launched before 2001 (n = 6) and higher (40.9 months) for those after 2001 (n = 14) (Table 2).

The proportion (%) of patients that reported having received blood and/or blood products varied from 32.9^45^ to 96.0%^33^. The weighted average for studies that provided this information (n = 15) was 74.3%; higher (75.4%) among studies launched before 2001 (n = 3) and lower (73.8%) for those after 2001 (n = 12) (Table 2).

**Figure 2:**
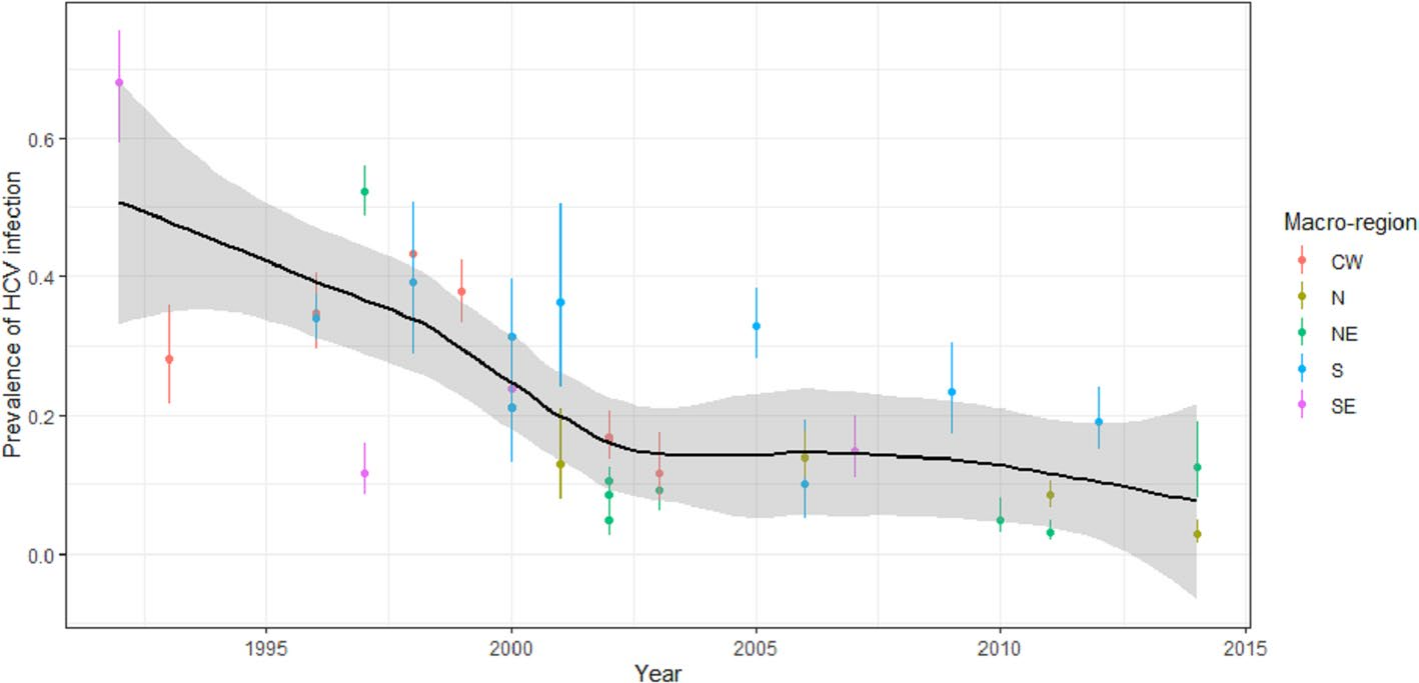
Prevalence rates of HCV infection and 95% credibility intervals in haemodialysis patients in Brazil from 1992 to 2015 according to major geographic region.

Prevalence of HCV infection (defined as a positive HCV antibody test) among the pool of studies on haemodialysis patients was highly heterogeneous (I^2^ = 98), with a pronounced decrease from 1992 to 2001, followed by a plateau and a slight decrease in recent years (Fig. 2).

Additional analyses presented in detail in the Web Appendix 3 show that such heterogeneities involve a complex combination of several factors, among them the year each study was carried out and the geographic region. These two factors are expected, given Brazil´s deep regional heterogeneity and the abovementioned decline of HCV prevalence over time.

But other factors are also key: studies summarise data from health services with heterogeneous clienteles, including small clinics as well as large referral centres, and there are relevant publication biases (as shown by the trim-and-fill statistics and the underlying funnel plots; Web Appendix 3).

As discussed below, nothing can be inferred about services which are not officially registered or for which the official registration has not been updated. There is no comprehensive and updated registry, precluding the matching of studies and the database partially updated by the surveys conducted by the Brazilian Society of Nephrology (as discussed below).

Finally, Baujat plots (Web Appendix 3) evidenced the influence of studies whose findings should be viewed as outliers. They were not excluded from the analysis, but rather analysed in detail in the abovementioned Appendix.

The summary measure for HCV prevalence was 34% (95% CI 26–43%) for studies implemented before 2001, with a high degree of heterogeneity (I^2^ = 96%). For studies launched after 2001, the corresponding summary measure was 11% (95% CI 8–15%), with persistently high heterogeneity (I^2^ = 95%).

**Figure 3:**
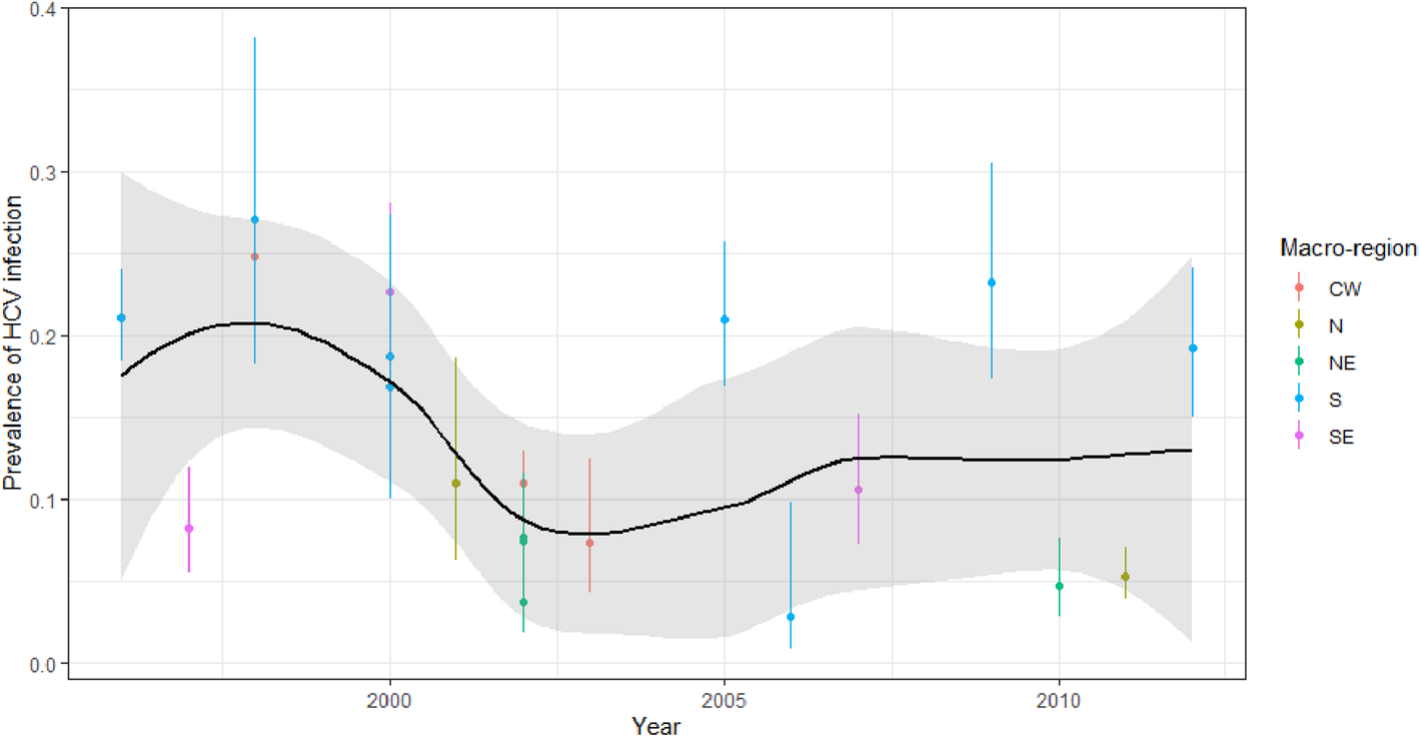
Prevalence rates for active HCV infection and 95% credibility intervals in haemodialysis patients in Brazil from 1996 to 2013 according to major geographic region of Brazil in which the studies were performed.

Estimates for prevalence of active HCV infection (i.e., patients with both positive HCV antibody and HCV-RNA results) were also highly heterogeneous (I^2^ = 94%). There was a marked decline from 1996 to 2001, followed by a plateau and a slight increase after 2010 (Fig. 3). The summary measure for active HCV infection was 19% (95% CI 15–25%) in studies launched before 2001, with a high degree of heterogeneity (I^2^ = 86%). For studies implemented after 2001, the corresponding summary measure was 9% (95% CI 6–13%), again with high heterogeneity (I^2^ = 93%) (Fig. 3).

The results from the random effects models fitted to data from studies implemented before 2001 (the cut-off we adopted for the entire period) for HCV prevalence (anti-HCV positivity) were as follows: 52% (95% CI 35–70%) in Northeast Brazil, 36% (95% CI 19–52%) in the Central-West, and 31% (95% CI 15–47%) in the South and Southeast. No studies were conducted in the North of Brazil in that period (Table 3).

**Table 3:** Results of binomial logistic models fitted to data from studies implemented before and after 2001 for HCV prevalence (positive HCV antibody test) and prevalence of active HCV infection (positive HCV antibody + HCV-RNA).

Macro-region	HCV prevalence (positive HCV antibody test)		Prevalence for active HCV infection (positive HCV antibody + HCV-RNA)	
	Before 2001	After 2001	Before 2001	After 2001
Northeast	52.3% (95% CI 48.7–55.8%)	7.9% (95% CI 6.9–8.9%)	NA	6.7% (95% CI 5.6–7.8%)
Central-West	37.7% (95% CI 35.1–40.4%)	16.1% (95% CI 14.4–17.9%)	24.8% (95% CI 20.8–29.0%)	10.5% (95% CI 8.9–12.3%)
South	33.1% (95% CI 30.4–36.0%)	25.1% (95% CI 22.3–28.0%)	20.9% (95% CI 18.5–23.4%)	19.3% (95% CI 16.7–22.0%)
Southeast	27.0% (95% CI 23.7–30.4%)	14.8% (95% CI 10.7–17.7%)	15.2% (95% CI 12.3–18.4%)	10.6% (95% CI 7.1–14.9%)
North	NA	8.7% (95% CI 7.4–10.1%)	NA	5.9% (95% CI 4.5–7.6%)

NA: No estimates were available, since no study was implemented in that time frame.

Prevalence of active HCV infection (i.e., positive HCV antibody + HCV-RNA) was as follows: 25% (95% CI 6–44%) in the Central-West, 21% (95% CI 3–38%) in the South, and 14% (95% CI 7–30%) in the Southeast. No estimates were available for the North or Northeast since no studies reported on the two tests in those regions during the period (Table 3).

The results from the random effects models fitted to data from studies implemented after 2001 for HCV prevalence were as follows: 23% (95% CI 9–33%) in the South, 15% (95% CI 3–27%) in the Central-West, 15% (95% CI 2–27%) in the Southeast, 8% (95% CI 4–18%) in the North, and 7% (95% CI 5–16%) in the Northeast. For active HCV infection, the results were as follows: 18% (95% CI 1–35%) in the South, 10% (95% CI 9–23%) in the Central-West, 11% (95% CI 7–24%) in the Southeast, 7% (95% CI 4–17%) in the North, and 6% (95% CI 5–17%) in the Northeast (Table 3).

The findings summarised in Web Appendix 4 show that some studies have information gaps and caveats. Statistical procedures were used to extract the best available information from them. Two senior authors (FIB and LMV) did their best to complement the available information by contacting authors, who, as a rule, are members of their network of peers. Some incremental information was obtained and included in Web Appendix 4.

Cross-comparison of the diagnostic tests used in the studies and the ANVISA spreadsheet on valid texts did not show any study using non-valid diagnostic kits or kits used after their official expiration, as defined by ANVISA.

## Discussion

Our study documented a substantial decline in HCV prevalence among Brazilian haemodialysis patients from 1992 to 2015. These findings are corroborated by data from the surveys conducted by the Brazilian Society of Nephrology (SBN in the Portuguese acronym). The SBN reported a pronounced decline from 1999 to 2018 with prevalence rates of 19.9% and 3.2%, respectively, in the network of clinics under the SBN umbrella^50,51^.

These are optimistic findings, roughly comparable to those of a recent study in the United States^52^. However, Brazil is a highly heterogeneous country, and results from pooled surveys of officially accredited clinics should be viewed with caution.

The surveys conducted by the SBN were not included in the current meta-analysis: (i) they are not first-hand empirical studies, but rather web surveys, and the haemodialysis clinics complete the e-forms on a voluntary basis, with no subsequent double-checking; (ii) haemodialysis and peritoneal dialysis are frequently pooled indistinguishably; and (iii) a progressive decrease in the proportion of services with valid responses has been observed over time, with a 37% response rate in 2018^51^.

Both the SBN web surveys and our own review were subject to biases, although to a much lesser extent in our review and meta-analysis. Empirical studies tend to be implemented in more accessible services with better infrastructure. Thus, neither our study nor the SBN surveys can properly overcome the limitation secondary to “invisible” services. Publication bias was made evident in Web Appendix 3. The trim-and-fill analysis allows to include hypothetical studies, depicted as blank circles.

Some services are simply excluded from any assessment and lack the necessary accountability. Such “invisible” services are likely to perform worse with the management of equipment and supplies. Therefore, they may have higher rates of various infections, including HCV. However, this limitation cannot be addressed fully without a comprehensive nationwide survey including all haemodialysis clinics, regardless of affiliation with the SBN.

For studies implemented from 2001 to 2015, the summary measures were 11% for HCV prevalence (95% CI 8–15%) and 9% for active HCV infection (95% CI 6–13%), with pronounced heterogeneity. For the same period, estimates of HCV prevalence made available by the Brazilian Society of Nephrology varied from 4 to 17%, with a progressive decline over time, unfortunately in tandem with a progressive decrease in the proportion of services that provided valid responses to the SBN web survey itself^50,53^.

It is essential to compare our findings with those of the single nationwide population-based survey on hepatitis C in Brazil. This probability survey, implemented in 2005–2009, found an overall estimate of 1.4% (95% CI 1.1–1.6%) in Brazil’s general population^54^. Such figures are substantially lower than our summary measure for the pool of studies implemented after 2001 (11%). Therefore, patients with CKD should be targeted with specific interventions to prevent HCV infection, and if prevention fails, prompt diagnosis and treatment.

There are relevant limitations that recommend caution when interpreting the findings of our systematic review as well as the summary measures of our meta-analysis:

i. The overall quality of original papers is far from optimal (as detailed in Web Appendix 4). Whatever the statistical analyses that might be used to address such a limitation, far from optimal quality of original data constitutes an insurmountable limitation;
ii. There is pronounced heterogeneity between (and within) Brazil’s five major geographic regions. Our study basically addressed interregional heterogeneities to avoid dealing with the small numbers in intraregional heterogeneities;
iii. There is a pronounced under-representation of the Southeast, which is the most densely populated and most industrialized region of Brazil. According to the National Censuses of 2000 and 2010 and estimates for 2020 by the IBGE (Brazilian Institute of Geography and Statistics), more than 40% of all Brazilians live in the Southeast^55^. This region concentrates roughly 50% of the country’s haemodialysis services^51^. In our study, only 12.5% of the studies were conducted in services located in the Southeast, corresponding to 8.0% of the overall sample. Such pronounced underestimation appears to be associated with a high degree of redundancy: whereas some referral services have been repeatedly assessed over time, a fraction of services remain “invisible”.
iv. As explained in detail in Web Appendix 1, ANVISA did not provide us with information on tests that might have been used in the field before (or without) the agency’s official authorization. However, this possibility is highly unlikely since use of unauthorized tests would be defined as a crime. Besides compromising any study’s integrity, it could lead to criminal prosecution of the perpetrators.
v. The available data probably underestimate the actual infection rates. Even third-generation immunoassays can yield false-negative results. The latter tend to be especially relevant among immunosuppressed patients, as frequently happens with CKD patients in haemodialysis, including low and/or intermittent viremia, besides poor antibody responses^56^.

Our estimates of HCV prevalence (after 2001) for the five major geographic regions of Brazil included those published by the Brazilian Society of Nephrology for 2002: Northeast (12%); Central-West (12%); South (20%); Southeast (15%); and North (12%)^50^. Both our study and the SBN survey highlight the South as the region of Brazil with the highest HCV infection rates in haemodialysis patients.

Considering the South of Brazil as a sentinel setting to target with preventive and/or curative interventions, it is important to note that HCV prevalence there (23%, 95% CI 9–38%) is comparable (with overlapping 95% CIs) to the prevalence rates in Iraq (20%; 95% CI 12–28%) and Turkey (23%; 95% CI 18–28%) and lower than the prevalence rates reported in Egypt (50%; 95% CI 46–55%) and Syria (54%; 95% CI 50–59%), according to a meta-analysis of studies conducted from 2006 to 2016^57^. For historical reasons, Egypt has the world’s highest HCV rates^58^, and Syria has been affected by a prolonged civil war, impacting the country’s overall infrastructure, including health services^59^.

A review of articles published before 1999 highlighted marked heterogeneity in a wide range of countries. Although it is difficult to infer consistent information for such a large, heterogeneous pool of countries, one can easily conclude that before the twenty-first century, HCV control in haemodialysis patients was far from optimal everywhere in the world, as shown by the following HCV prevalence rates: 4–14% in the UK, 5–10% in Denmark, 12% in Sweden, 12% in India, 4–23% in Germany, 14%, 5–44% in the USA^60^. Rates as high as 71% in Kuwait or exceeding 40% in a long list of countries should obviously be defined as a totally uncontrolled situation and as a major source of new HCV infections. And this public health disaster struck when treatment was far from adequate.

With strict adherence to biosafety standards concerning equipment, supplies, and health workers and ancillary staff, most high-income and some middle-income countries have curbed various infections in haemodialysis patients. In Brazil, the impact of such procedures appears to be mixed, with some referral centres presenting infection rates comparable to high-income countries, whereas other centres present persistently high rates of various infections, including HCV. The lack of comprehensive multi-centre studies precluded a careful assessment of services located in the most deprived areas or the adoption of sound policies^61^.

A substantial decrease has been observed, still far from optimal. Improvements have been uneven, with major advances in some services and no discernible improvement in others, where even the most basic information is simply lacking.

Unfortunately, with the emergence of COVID-19 and the inadequate response by the Brazilian government^62^ most health services, including haemodialysis centres, faced major disruptions. The effects of the epidemic have been combined with a deep political and economic crisis^62^, with a shrinking budget and permanent threats to democratic institutions, such as the Supreme Court^63^, and the legislation and norms issued by them.

Although far from systematic, some preliminary evaluations published in 2020^11^ as well as anecdotal reports by the media^64^ suggest that we may have been facing a “perfect storm”. These unfortunate events may compromise and even reverse the modest gains obtained so far in the prevention, management and care of hepatitis C among patients in haemodialysis.

Although the goal of HCV elimination by 2030 in Brazil appears elusive, especially in the context of the COVID-19 pandemic and budget restrictions^65^, it is possible and necessary to adopt measures to achieve micro-elimination in some settings and to launch initiatives towards targeted interventions to curb the spread of HCV in people with CKD, among other high-risk groups. Given the current situation, this will require a sound advocacy coalition^66^ comprising the scientific community, health professionals, policymakers, committed politicians, and civil society.

A comprehensive combination of preventive and curative initiatives should be adopted: prevention can be achieved through safer, state-of-the-art procedures^67^, timely diagnosis and referral, and drug regimens with direct-acting antivirals (DAAs) presenting low levels of nephrotoxicity^68^. The literature has documented this as a feasible goal. Patients with CKD in haemodialysis units can and must be properly managed to prevent HCV infection; in case they become infected, they can and must be cured of hepatitis C, as recently reviewed by Brazilian researchers^69^.

As of 2018, there were 123,187 patients in Brazil with CKD who were undergoing haemodialysis^51^. The lack of proper surveillance and policies to curb the spread of HCV and of prompt management and treatment tends to be a key source of sustained transmission and unnecessary suffering and avoidable deaths.

## Supplementary Information

Supplementary Information 1.

Supplementary Information 2.

Supplementary Information 3.

Supplementary Information 4.

Supplementary Information 5.

Supplementary Information 6.
