# Supplementary material for: Persistently high hepatitis C rates in haemodialysis patients in Brazil [a systematic review and meta-analysis]
Source: Sci Rep. 2022 Jan 10;12:330. doi: 10.1038/s41598-021-03961-x (PMC8748660; doi:10.1038/s41598-021-03961-x)
Supplement: Supplementary file 1 — Supplementary Information 1. [file 41598_2021_3961_MOESM1_ESM.pdf]

Search strategies

A preliminary search and extraction were performed by three authors of the current article (RPN, DCMB, and JSF), using a blinded procedure and the search terms defined in the article´s original version.

In agreement with the requirements by PROSPERO and the suggestions submitted to the authors by an anonymous reviewer, search strategies were redefined and substantially broadened. Entirely new searches were performed, as well as a new round of extraction. This second and final procedures were performed independently by two other authors (JCM and FIB). The second search and extraction allowed the authors to double-check previous findings and to implement a comprehensive quality assessment of the articles.

We purposely avoided placing a limitation on the final year under analysis in this step.

The search strategies were redefined with the inclusion of new terms, suggested by both the reviewer and us and in agreement with the MeSH controlled vocabulary [https://www.ncbi.nlm.nih.gov/mesh/], as well as after suggestions adopted by The Cochrane Initiative, Brazil office [https://brazil.cochrane.org/o-centro-cochrane-do-brasil]. The latter highlights problems that are specific to Brazil such as the comprehensive list of the country´s states and geographic regions.

The new equations were applied to the same databases: PubMed; LILACS; Web of Science [Core Collection] & SCOPUS.

Despite the non-inclusion of 2020 in the final analyses for reasons explained in the main body of the text, all exploratory analyses included 2020.

This decision was intentional: we looked for potential indexation errors and browsed each of the new articles vis-à-vis the comprehensive list of valid tests. At request, the list was provided by ANVISA (the Brazilian Health Regulatory Agency) (# ANVISA Protocol 2021195961, September 3, 2021). ANVISA provided a list of all validated hepatitis C (HCV) tests in Brazil, with their respective expiration dates. Additional information was compiled as a comprehensive e-spreadsheet which is being shared with our potential readers in a specific new Web appendix.

It is essential here to address the misuse of test kits after their expiration dates, as in the use of batches of expired kits. This practice is a misdemeanour. Of course, it would invalidate any study in which such tests were used. On the other hand, ANVISA failed to inform us about the putative importation and use of tests before its authorization. This is defined by Brazilian law as not as a misdemeanour, but rather as a felony − illegal importation and contraband of illicit materials. ANVISA may release such information after a joint formal request to the agency and the Federal Police.

Although this may constitute a limitation of our study, since illegal importation and misuse of tests can happen anywhere in the world, the issue is beyond the scope of our review, which has nothing to do with criminal justice.

The new search yielded a total of 34 studies, 8 of which from 2020, 3 from 2019, 2 from 2018, 3 from 2016, and 19 published from 1994 to 2015.

An exception was made for 2020, where exclusions were due to the profound changes and major disruptions from the COVID-19 pandemic on all health services, and all other additional studies were excluded for not having met the inclusion criteria.

The new search equations are described as follows:

SCOPUS (Elsevier host; year < 2020; search hits = 104)

TITLE-ABS-KEY((Prevalence OR seroprevalence OR Incidence OR rate OR occurrence OR frequency OR Epidemiology OR diagnosis OR mortality OR "prevention and control" OR "statistical and numerical data" OR transmission) AND ("Hepatitis C" OR HCV OR "liver disease") AND (Hemodialysis OR dialysis OR “Renal Dialysis”) AND (Brazilian OR Brazil OR Acre OR Alagoas OR Amapá OR Amazonas OR Bahia OR Ceará OR “Distrito Federal” OR “Espírito Santo” OR Goiás OR Maranhão OR “Mato Grosso” OR “Mato Grosso do Sul” OR “Minas Gerais” OR Pará OR Paraíba OR Paraná OR Pernambuco OR Piauí OR “Rio de Janeiro” OR “Rio Grande do Norte” OR “Rio Grande do Sul” OR Rondônia OR Roraima OR “Santa Catarina” OR “São Paulo” OR Sergipe OR Tocantins)) AND (PUBYEAR < 2021)

MEDLINE (1989/01/01 to 2020/12/31; search hits = 57)

((Prevalence[Title/Abstract] OR seroprevalence [Title/Abstract] OR Incidence [Title/Abstract] OR rate [Title/Abstract] OR occurrence [Title/Abstract] OR frequency [Title/Abstract] OR Epidemiology [Title/Abstract]) AND (“Hepatitis C”[Title/Abstract] OR HCV[Title/Abstract] OR “liver disease”[Title/Abstract] OR “Hepatitis C” [MeSH Terms] OR “liver disease” [MeSH Terms]) AND (Hemodialysis[Title/Abstract] OR Dialysis [Title/Abstract] OR “Renal Dialysis” [MeSH Terms]) AND (Brazilian[Title/Abstract] OR Brazil[Title/Abstract] OR Acre [Title/Abstract] OR Alagoas [Title/Abstract] OR Amapá [Title/Abstract] OR Amazonas [Title/Abstract] OR Bahia [Title/Abstract] OR Ceará [Title/Abstract] OR “Distrito Federal” [Title/Abstract] OR “Espírito Santo” [Title/Abstract] OR Goiás [Title/Abstract] OR Maranhão [Title/Abstract] OR “Mato Grosso” [Title/Abstract] OR “Mato Grosso do Sul” [Title/Abstract] OR “Minas Gerais” [Title/Abstract] OR Pará [Title/Abstract] OR Paraíba [Title/Abstract] OR Paraná [Title/Abstract] OR Pernambuco [Title/Abstract] OR Piauí [Title/Abstract] OR “Rio de Janeiro” [Title/Abstract] OR “Rio Grande do Norte” [Title/Abstract] OR “Rio Grande do Sul” [Title/Abstract] OR Rondônia [Title/Abstract] OR Roraima [Title/Abstract] OR “Santa Catarina” [Title/Abstract] OR “São Paulo” [Title/Abstract] OR Sergipe [Title/Abstract] OR Tocantins [Title/Abstract] OR Brazil [MeSH Terms]) AND ("1989/01/01"[Date - Publication] : "2020/12/31"[Date - Publication])

Note: The period was then narrowed to the beginning of 1989, when the very concept of hepatitis C replaced the former concept of “non A/non B” Hepatitis (apud Pawlotsky J-M. J Hepatol 2015; 62(1 Suppl):S87-99, doi: 10.1016/j.jhep.2015.02.006). Searches comprising the term “non-A non-B hepatitis comprised a hypothetical majority of HCV infections, but this at the cost of a high degree of “noise”, such as other hepatitides, about which there is no accurate re-assessment.

LILACS (Bireme; 1989 to 2020) (search hits = 103)

(Prevalência OR Prevalencia OR Prevalence OR Soroprevalência OR seroprevalencia OR seroprevalence OR Incidência OR incidência OR Incidence OR Taxa OR tasa OR rate OR Epidemiology OR Epidemiologia OR diagnosis OR diagnóstico OR mortality OR mortalidade OR transmission OR transmissão) AND ("Hepatite C" OR "Hepatitis C" OR VHC OR HCV OR "doença hepática" OR "enfermedad del hígado" OR “liver disease”) AND (Hemodiálise OR hemodiálises OR Hemodialysis OR Diálise OR Diálisis OR Dialysis OR “Renal Dialysis” OR “Diálise Renal”) AND (Brasil OR Brazil OR Brasileiro OR Brasileira OR Brazilian OR Acre OR Alagoas OR Amapá OR Amazonas OR Bahia OR Ceará OR "Distrito Federal" OR "Espírito Santo" OR Goiás OR Maranhão

OR "Mato Grosso" OR "Mato Grosso do Sul" OR "Minas Gerais" OR Pará OR Paraíba OR Paraná OR Pernambuco OR Piauí OR "Rio de Janeiro" OR "Rio Grande do Norte" OR "Rio Grande do Sul" OR Rondônia OR Roraima OR "Santa Catarina" OR "São Paulo" OR Sergipe OR Tocantins) [Palavras] AND "1989" OR "1990" OR "1991" OR "1992" OR "1993" OR "1994" OR "1995" OR "1996" OR "1997" OR "1998" OR "1999" OR "2000" OR "2001" OR "2002" OR "2003" OR "2004" OR "2005" OR "2006" OR "2007" OR "2008" OR "2009" OR "2010" OR "2011" OR "2012" OR "2013" OR "2014" OR "2015" OR "2016" OR "2017" OR "2018" OR "2020" [País, ano de publicação]

Web of Science (1989 to 2020) (search hits = 71)

Topic: (((Prevalence OR seroprevalence OR Incidence OR rate OR occurrence OR frequency OR Epidemiology OR diagnosis OR mortality OR "prevention and control" OR "statistical and numerical data" OR transmission) AND ("Hepatitis C" OR HCV OR "liverdisease") AND (Hemodialysis OR dialysis OR “Renal Dialysis”) AND (Brazilian OR Brazil OR Acre OR Alagoas OR Amapá OR Amazonas OR Bahia OR Ceará OR Distrito Federal OR Espírito Santo OR Goiás OR Maranhão OR Mato Grosso OR Mato Grosso do Sul OR Minas Gerais OR Pará OR Paraíba OR Paraná OR Pernambuco OR Piauí OR Rio de Janeiro OR Rio Grande do Norte OR Rio Grande do Sul OR Rondônia OR Roraima OR Santa Catarina OR São Paulo OR Sergipe OR Tocantins)))

Refined by: year of publication: from 1989 on, as previously explained.

Search strategy: Steps and procedures

The first step was a detailed reading by independent reviewers of the titles of all articles selected with the search algorithm. Articles with no clear link to the study’s purpose and inclusion criteria were excluded before any further steps.

The second step consisted of the analysis of the contents of abstracts from all the articles approved in step 1. Abstracts were screened for their relevance to the study’s objectives and criteria. When the abstracts/articles were consistent with these criteria, the full texts were read by the reviewers.

The reviewers extracted core information from the articles selected for full-text reading, using a standard form completed independently by each of the three reviewers. The standard form included the following variables: name of the first author; the year(s) the study was implemented and concluded; the major geographic region of Brazil (among five) where the study was conducted; the target population/patient group; sample size; laboratory tests used for diagnosis of HCV infection; prevalence of HCV infection; proportion (%) of individuals who had ever received blood/blood products; average age (in years) of the haemodialysis patients; and average time on haemodialysis. Averages were defined as arithmetic means. In the absence of means, the medians or interpolated values were used.

The fourth and final step consisted of the application of the exclusion criteria to all the articles. When the three independent reviewers failed to reach the same decision, the article was discussed until a consensus was reached.

Basic assumptions of statistical analyses

The analyses and corresponding graphs made the following assumptions: i) the number of HCV-positive patients in each study *Y* followed a binomial distribution, with a parameter *n* corresponding to the number of patients tested in the context of each study and ii) the *a priori* distribution was also assumed as uniform (0, 1) for the different studies.

The subsequent distribution of studies was defined as a beta (β) distribution (y+1,n-y+1). The 95% credibility intervals were calculated based on this distribution.

The graphs were produced by fitting a local polynomial regression (*loess*), taking the year each study was launched and *a posteriori* mean prevalence as the predictor.
