# Supplementary material for: Persistently high hepatitis C rates in haemodialysis patients in Brazil [a systematic review and meta-analysis]
Source: Sci Rep. 2022 Jan 10;12:330. doi: 10.1038/s41598-021-03961-x (PMC8748660; doi:10.1038/s41598-021-03961-x)
Supplement: Supplementary file 2 — Supplementary Information 2. [file 41598_2021_3961_MOESM2_ESM.pdf]

CoCoPop – **Co**ndition, **Co**ntext and **Po**pulation

CoCoPop, similar to PICO (Population, Intervention, Comparation and Outcome) and PEO (Population, Exposure and Outcome), precedes the bibliographic search and guides the definition of the keywords.

It provides a conceptual and operational framework for the research question, as summarised by the acronym "CoCoPop", guiding the construction of the search equations. CoCoPop is used in systematic reviews and meta-analysis on prevalence or incidence, in cases where there is no interventions or comparisons (Munn et al., 2018; Pollock and Berge, 2018).

Patients with any severe medical condition, as is the case of people with chronic kidney disease, usually take different medicines and may need additional interventions, such as rehydration. Notwithstanding, there is no clear association of such procedures and hepatitis C. None of the papers reviewed by our group mentioned such procedures or have made any attempt to control their findings for putative risks associated with them. For the sake of our study, haemodialysis is an inclusion criterion and no additional intervention was included in the analysis, both due to the absence of any empirical information or insights from the literature.

Research question: *What is the prevalence of the infection by Hepatitis C in h(a)emodialysis* patients in Brazil?* [British/American English]

Table S1. Components of the research question, according to the anagram CoCoPop

Description	Abbreviation	Components
**Co**ndition	Co	Infection by Hepatitis C
**Co**ntext	Co	Hemodialysis in Brasil (XXXX* to 2019) and infection by Hepatitis C
**Pop**ulation	Pop	Haemodialysis patients in Brazilian healthcare units, independent of gender or age

* There was/is no a priori definition of the commencement of the period. Of course, it depends on: i) the implementation of haemodialysis in Brazil and of assessments of services providing such services for patients in need; ii) the clear definition of hepatitis C, replacing the former fuzzy category "non A/non B hepatitis"; iii) the availability and full use of diagnostic tests with acceptable accuracy, as defined in the body of the text.

Table S2. Application of CoCoPop in the definition of search terms

Population	Context Year	Context Hemodialysis	Context Infection	Context Country	Condition
Haemodialysis patients in Brazilian healthcare units, independent of gender or age.	XXXX to 2019	Hemodialysis; Hemodiálise; hemodiálises; Diálise; Diálisis; Dialysis; Renal Dialysis; Diálise Renal	Prevalence; Prevalência; Prevalencia; Soroprevalência; seroprevalence; Incidence; Incidência; Taxa; tasa; rate; occurrence;	Brazil; Brasileiro; Brasileira; Brazilian; Acre; Alagoas; Amapá; Amazonas; Bahia; Ceará; "Distrito Federal"; "Espírito Santo"; Goiás; Maranhão;	"Hepatitis C"; HCV; "Hepatite C"; VHC; "doença hepática"; "enfermedad del hígado"; "liver disease"

Population	Context Year	Context Hemodialysis	Context Infection	Context Country	Condition
			ocorrência; frequency, frequência; Epidemiology, Epidemiologia; diagnosis; diagnóstico; mortality; mortalidade; prevention and control; statistical and numerical data; transmission; transmissão	"Mato Grosso"; "Mato Grosso do Sul"; "Minas Gerais"; Pará; Paraíba; Paraná; Pernambuco; Piauí; "Rio de Janeiro"; "Rio Grande do Norte"; "Rio Grande do Sul"; Rondônia; Roraima; "Santa Catarina"; "São Paulo"; Sergipe; Tocantins	

Bibliography:

Munn Z, Stern C, Aromataris E, Lockwood C, Jordan Zoe. What kind of systematic review should I conduct? A proposed typology and guidance for systematic reviewers in the medical and health sciences. BMC Medical Research Methodology 2018; 18:5, DOI 10.1186/s12874-017-0468-4

Pollock A, Berge E. How to do a systematic review. International Journal of Stroke 2018; 13(2):138-156, DOI: 10.1177/1747493017743796
