# Supplementary material for: Persistently high hepatitis C rates in haemodialysis patients in Brazil [a systematic review and meta-analysis]
Source: Sci Rep. 2022 Jan 10;12:330. doi: 10.1038/s41598-021-03961-x (PMC8748660; doi:10.1038/s41598-021-03961-x)
Supplement: Supplementary file 3 — Supplementary Information 3. [file 41598_2021_3961_MOESM3_ESM.pdf]

Summary

1. Overall explanations on the use of forest plots, I^2 statistics, meta-regression, trim-and-fill, and analyses of the putative influence of different effects (sample size, publication year) 2

2. Findings 5

2.1 HCV infection 5

2.1.1. Forest plots and respective tests 5

2.1.2. Subgroup analysis 6

2.1.3. Meta-regression 7

2.1.4. Trim-and-fill 9

2.1.5. Influence analysis 9

2.2 Hepatitis C prevalence 11

2.2.1. Forest plot Q, heterogeneity, and I^2 statistics 11

2.2.2. Subgroup analysis 12

2.2.3. Meta-regression 13

2.2.4. Trim-and-fill 14

2.2.5. Influence analysis 15

1. Overall explanations on the use of forest plots, I^2 statistics, meta-regression, trim-and-fill, and analyses of the putative influence of different effects (sample size, publication year)

(1.a) *Forest plot:* Forest plots depict the distribution of prevalence for each of the studies, as well as the summary measure.

There are two vertical lines on the Y axis, one corresponding to the summary measure (prevalence) of the **fixed effects model (FEM)** and the other corresponding to the summary measure generated by the **random effects model (REM)**.

FEM assumes that prevalence of each of the studies is similar and comparable (to other studies), and that the observed differences are secondary to sampling errors (within-study variability). In other words, the model assumes that the observed between-studies variability does not follow any discernible pattern and can be defined for practical purposes as random.

Meanwhile, REM assumes that prevalence is NOT similar between different studies. Rather, the different studies extract different information from a hypothetical all-encompassing set of studies.

The distance between each study outcome and the vertical axes allows visualizing their discrepancy vis-à-vis the summary measure.

Two small diamond-shaped figures at the bottom of the graph are the visual equivalents of the pooled prevalence rates and respective CIs generated by the FEM and REM. As easily observed, REM yields larger CIs since smaller samples have a larger share in the pooled analysis. There is a trade-off here: CIs generated by FEM enjoy better accuracy, while accepting overly relaxed assumptions. The latter have been violated frequently under real-life conditions.

(1.b) *Assessing heterogeneity:* The different statistics are as follows: tau^2, χ^2 (chi-square), and I^2 statistics document the extent to which studies are similar or different, that is, homogeneous or heterogeneous. Overly high heterogeneity means summary measures are far from optimal (in some cases, they may preclude any meaningful synthesis).

In case the REM is used, the tau^2 statistic corresponds to the variance of the outcome under analysis and the between-studies variability. The χ^2 statistic is traditionally used for the same purpose. It usually adopts 0.05 as the standard cut-off for defining heterogeneity (recent studies have criticized its use as an exclusive way to define statistical significance [https://www.amstat.org/asa/files/pdfs/p-valuestatement.pdf]. We interpret it with the necessary caution and in tandem with the other statistics.

The I^2 statistic varies from 0% to 100% and should be interpreted as follows: 0% corresponds to a hypothetical perfect homogeneity; 0%-25% should be interpreted as low heterogeneity; ~50% as moderate heterogeneity, and >75% as pronounced heterogeneity. The I^2 statistic was chosen as a measure of heterogeneity because it is more consistent, compared to Cochran's Q test (Higgins et al., 2003) for purposes of

meta-analyses comprising few studies and/or with studies with small sample sizes. This scenario has been observed in most studies (Hoaglin, 2016).

(1.c) *Subgroup analysis*: Subgroup analysis can help elucidate the source(s) of heterogeneity. The procedure basically consists of the generation of multiple forest plots and respective statistics for different subgroups of studies belonging to a given set. It is useful here to distinguish the notion of 'qualitative interaction' from that of 'quantitative interaction' between different study subgroups (Yusuf et al., 1991). Useful advice concerning subgroup analyses is available in Oxman & Guyatt (1992) and Yusuf et al. (1991).

(1.d) *Meta-regression*: Different covariates are repeatedly tested in a specific model for purposes of better discerning whether one or more covariates may play a key role vis-à-vis the observed variability. The procedure has some limitations such as the minimum number of studies for each covariate to be putatively inserted or excluded (usually assumed as ≥10 different studies). The second limitation is the need to use a full covariance matrix, which is heavily affected by missing values.

For the present study´s purposes, simple and multivariate models were fitted, taking Brazil's major geographic regions and the year the studies have been carried out into consideration. The respective estimates under consideration in our models were I^2 (defined here as the ratio between the residual heterogeneity of the models and the variability not accounted for), the test of moderators using the Fisher-Snedecor F distribution. The F distribution allows assessing whether a given predictor (covariate) has been statistically associated with the differences between prevalence for the different studies, besides calculating the 95% confidence interval associated with each regression coefficient. The model chosen for this analysis is REM, after a logit transformation of the point prevalence.

(1.e) *Trim-and-fill*: Trim-and-fill has been used to assess (and occasionally adjust) the potential publication bias (Duval, 2005).

The graphic basis of trim-and-fill is provided by the funnel plot, a dispersion graph plotting the studies´ size effect on the X axis versus the standard errors in the Y axis. The graph depicts the most accurate studies at the top and the least accurate ones at the base of the pyramid. Small studies´ estimates tend to be concentrated near the pyramid´s base.

In the absence of discernible bias, the studies (depicted as dots) tend to be scattered, roughly in the shape of an inverted pyramid. The trim-and-fill procedure moves beyond visualization, providing a Q statistic that can provide a quantitative basis for the assessment of potential biases secondary to publication heterogeneity. Eggers' test was also calculated. Conceptually, it is roughly equivalent to the Q statistic (Egger et al. 1997).

In the graph, the small solid circles represent the actual studies included in the meta-analysis, whereas the blank circles correspond to studies that are potentially absent but

have been imputed by the procedure. The dashed lines define the triangular area corresponding to the 95% CIs, and the vertical line depicts the overall effect.

(1.f) *Influence analysis*: Influence analysis aims to assess whether one or more studies may have a substantial influence on the summary measure and/or increase the overall heterogeneity.

One or several characteristics of each study are inspected to evaluate their putative influence. Baujat plot is a standard way to visualize such putative influences. It is fully available in the respective R library (https://www.rdocumentation.org/packages/meta/versions/4.9-6/topics/baujat). The X axis of the Baujat plot depicts the contribution of a given study vis-à-vis the overall heterogeneity, whereas the Y axis depicts the influence of the size effect of a given study versus the overall effect (weighted by the inverse of the variance) (Baujat et al., 2002).

Studies presenting high values in the two axes are defined as having a pronounced influence on the summary measures of the meta-analysis, that is studies that influence the measures due to their impact on both heterogeneity and overall effect. The same interpretation applies to different combinations of values on both the X and Y axes.

A second graph was plotted to assess the influence of the sample size of each study vis-à-vis the summary measures for both HCV infection and hepatitis C.

Overall, this second graph depicts the relationship between the prevalence of each study, its corresponding sample size, and the estimates (with their respective 95% confidence intervals) of the predicted prevalence rates resulting from the meta-regression (Borenstein et al., 2009).

Studies with large samples where a low prevalence was observed or vice-versa may exist due to the very nature of the different facilities (for instance, large and specialized clinics versus small, understaffed services) and the characteristics of the clientele seeking or referred to a given service (for instance, referral services tend to concentrate complex cases that cannot be managed properly by less specialized services; An et al., 2018).

Visual inspection allows identifying potential outliers (that is, points located far from the main area where most studies tend to cluster) or studies which can exert a pronounced influence on the summary measures.

Using two successive graphs, we assessed the influence of sample size and publication year, respectively. In addition to visualizing the effect of the studies´ sample size on prevalence rates, it is possible to observe the differences between observed and predicted prevalence rates, as well as to examine the potential influence of different independent variables (e.g., sample size and the year each study was carried out (not the year of publication of the respective peer-reviewed paper).

2. Findings

2.1 HCV infection

2.1.1. Forest plots and respective tests

Figure 1 shows studies assessing HCV infection. The forest plot helps visualize the heterogeneity described in the main body of the text (the scattered distribution around the vertical axes). The study by Oliveira et al. (2001) should be viewed as a potential outlier. Heterogeneity has been assessed by different statistics, as summarized in **Chart 1** below:

Figure 1. Forest plot of studies on HCV prevalence

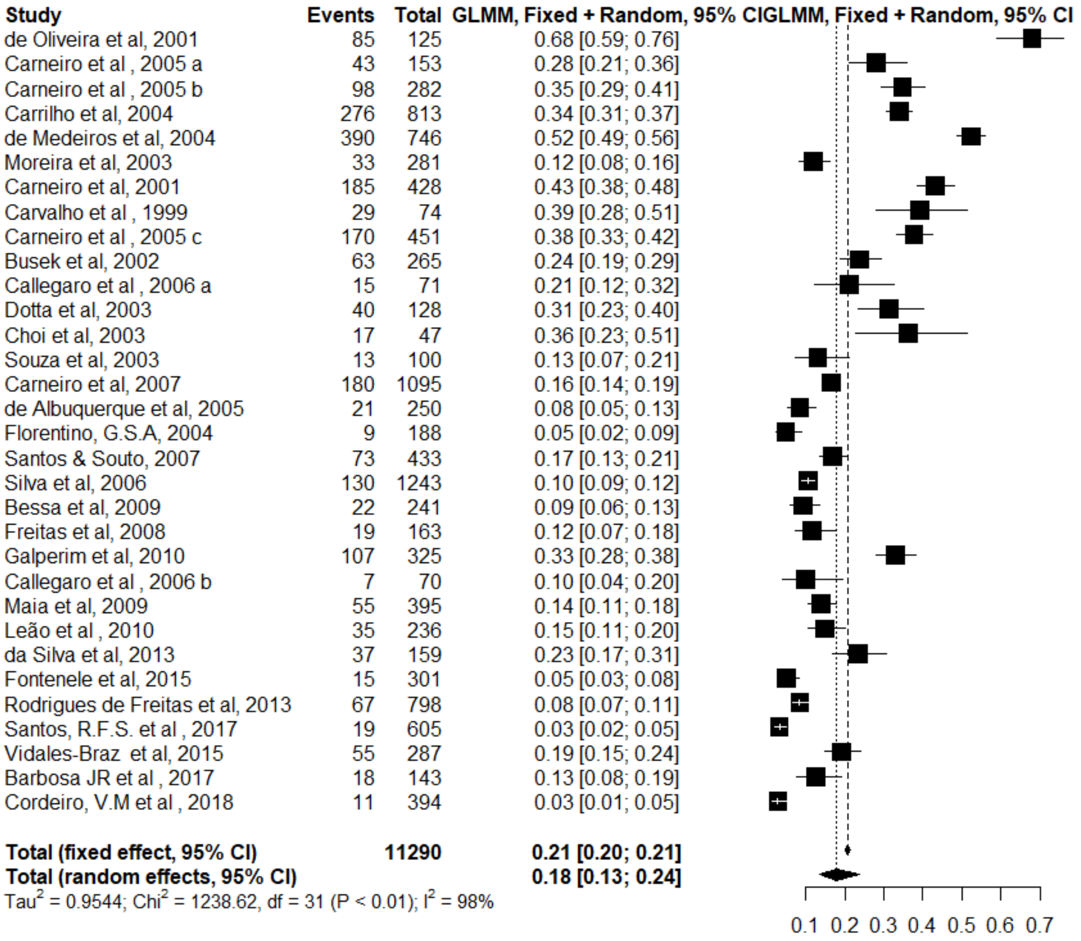

Chart 1. Heterogeneity assessment by different statistics (n=32)

Random effects model = 0.1790; 95%CI [0.1337 – 0.2356]

Chi-square = 1238.62; df = 31; p-value: <0.01

I^2 = 98%

Cochran Q Test:

Q	d.f.	p-value	Test
1238.62	31	< 0.0001	Wald-type
1520.60	31	< 0.0001	Likelihood-Ratio

Figure 1.a shows studies assessing HCV infection by the year each study was carried out. The forest plot helps to visualize heterogeneity, stratified by year (< 2001 and >= 2001).

Figure 1.a. Forest plot of studies on HCV prevalence by year

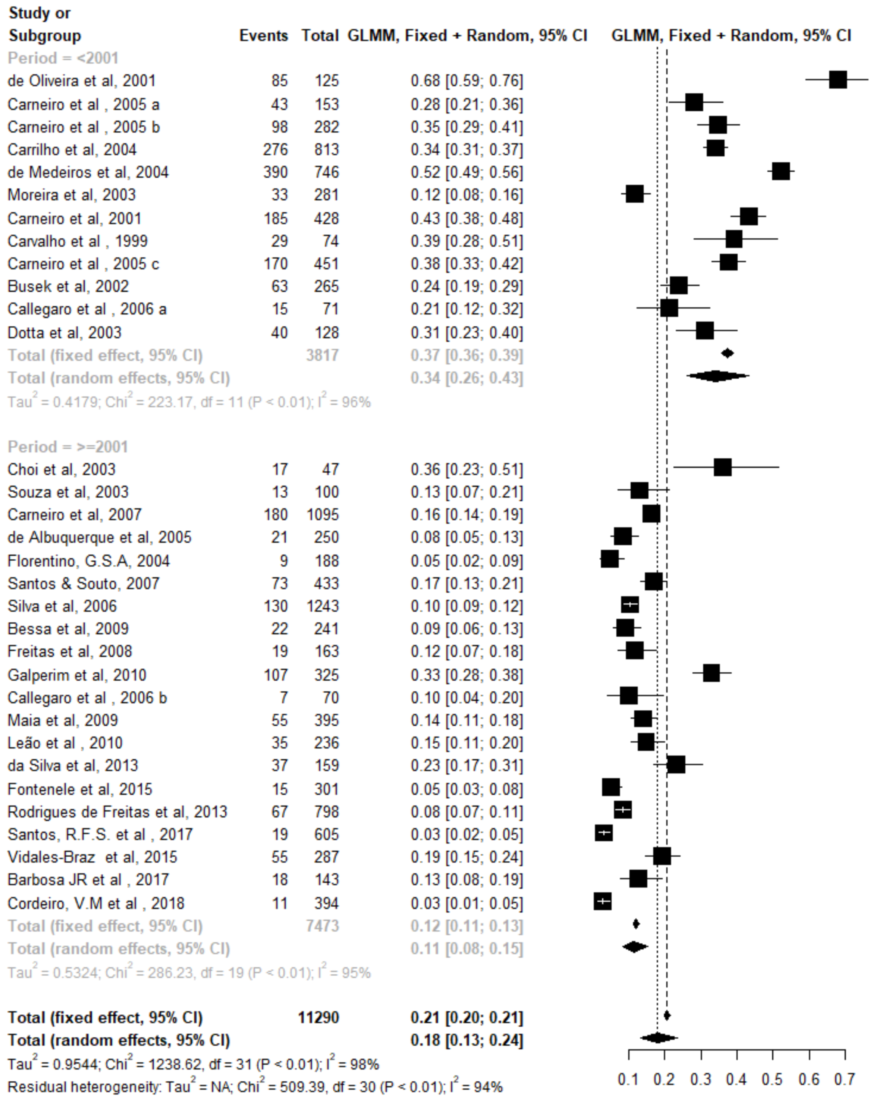

2.1.2. Subgroup analysis

Table 1a summarize the findings from the subgroup analysis by major geographic regions of Brazil. Summary prevalence rates and respective 95%CIs are stratified by major geographic regions of Brazil. Cochran Q Test had a p-value <0.001, refuting the null hypothesis (that is. prevalence did not differ according to major geographic region).

Table 1a. Subgroup analysis for the Brazil´s major geographic regions

Region	K (studies)	Prevalence [95%CI]	tau^2
Southeast	4	0.2590 [0.1076; 0.5032]	1.1480
Central-West	7	0.2544 [0.1781; 0.3496]	0.3558
South	9	0.2682 [0.2138; 0.3306]	0.1608
Northeast	8	0.0948 [0.0485; 0.1770]	1.0248
North	4	0.0822 [0.0446; 0.1466]	0.3897

Test for subgroup differences (random effects model):

	Q	d.f.	p-value
Between groups	22.94	4	0.0001

Table 1b summarize the findings from the subgroup analysis by the year each study was carried out. Summary prevalence rates and respective 95%CIs are stratified by year. Cochran Q Test had a p-value <0.001, refuting the null hypothesis (i.e. prevalence did not differ according to the year the studies were carried out).

Table 1b. Subgroup analysis for the year of the studies

Year	K (studies)	Prevalence [95%CI]	tau^2
< 2001	12	0.3423 [0.2630; 0.4316]	0.4179
>= 2001	20	0.1144 [0.0846; 0.1529]	0.5324

Test for subgroup differences (random effects model):

	Q	d.f.	p-value
Between groups	29.32	1	< 0.001

2.1.3. Meta-regression

Table 2a shows the findings from the meta-regression for HCV infection by major geographic region. The North and Northeast regions of Brazil were the main drivers of heterogeneity. The differences between the estimates, stratified by geographic regions, were statistically significant at p-value <0.000.

Table 2a. Findings from the meta-regression for HCV infection, by major geographic region

Region	Coefficient model			Predict[a]	Estimated prevalence [b]			P-value
	Coefficient	95% CI			Prevalence	95% CI		
		LL	HL			LL	HL	
North	-1.338	-2.316	0.361	-2.419	8.17	4.85	17.66	0.0073
Northeast	-1.161	-1.964	-0.358	-2.242	9.60	4.56	19.81	0.0046
South	0.051	-0.731	0.833	-1.029	26.32	11.06	41.60	0.8974
Southeast	0.028	-0.938	0.995	-1.052	25.88	10.70	41.05	0.9543
Central-West	-1.081	-1.661	-0.500	-1.081	25.34	10.27	40.41	0.0003

Notes: intercept model $\beta_0 =$ -1.0806 [a] $Predict = \beta_0 + \beta_1 Region$ [b] $Estimated\ prevalence = \frac{1}{1 + e^{[-(predict)]}}$

Heterogeneity: I^2 = 96.2903%; Test of Moderators: QM (df = 4) = 18.4765, p-value = 0.0010

Briefly, the studies conducted in two geographic regions found inverse associations with HCV infection: North (-1.3387; 95%CI [-2.316; 0.3611] and Northeast (-1.1613; 95%CI [-1.9643; -0.3582]). Cross-comparing them with the estimated overall HCV prevalence, using **REM** (**Figure 1**), both the North and Northeast showed significantly lower

prevalence than the summary estimate (18%; 95%CI:13%-24%) (**Figure 1**). Summary measures are influenced by such regional heterogeneities.

Table 2b shows the findings from the meta-regression for HCV infection by the year each study was carried out. The differences between the estimates, stratified by year, were statistically significant (p-value <0.000).

Table 2b. Findings from the meta-regression for HCV infection by year

Year	Coefficient model			Predict[a]	P-value
	Coefficient	95% CI			
		LL	HL		
< 2001	-0.654	-1.060	-0.248	-0.654	0.0016
>= 2001	-1.391	-1.909	-0.874	-2,046	<0.001

Notes: intercept model $\beta_0 =$ -0,6541 [a] $Predict = \beta_0 + \beta_1 Year$

Heterogeneity: I^2 = 95.7941%; Test of Moderators: QM (df = 1) = 27.7729, p-value = <0.0001

The same applied when meta-regression was stratified by study year (**Table 2b**). Studies conducted after 2001 showed significantly lower HCV prevalence than the previous ones, as discussed in the body of the article.

Table 2c shows the findings from the meta-regression for HCV infection of the studies implemented before and after 2001 by major geographic region. The differences between the estimates, stratified by year and major geographic region, were statistically significant for studies carried out after 2001 (p-value <0.000).

Table 2c. Meta-regression for HCV infection by year and major geographic region

Year	Region	Coefficient model			Predict[a]	Estimated prevalence [b]			P-value
		Coefficient	95% CI			Prevalence	95% CI		
			LL	HL			LL	HL	
Before 2001	North	NA	-	-	-	-	-	-	-
	Northeast	0.674	-0.634	1.983	0.091	52.28	34.98	69.59	0.3123
	South	-0.215	-1.068	0.638	-0.798	31.06	15.02	47.08	0.6219
	Southeast	-0.238	-1.152	0.676	-0.821	30.56	14.56	46.52	0.6100
	Central-West	-0.583	-1.176	0.009	-0.583	35.82	19.21	52.43	0.0537
After 2001	North	-0.660	-1.359	0.039	-2.397	8.34	4.46	17.91	0.0643
	Northeast	-0.849	-1.480	-0.218	-2.587	7.00	4.91	15.84	0.0083
	South	0.550	-0.119	1.219	-1.188	23.37	8.71	38.03	0.1069
	Southeast	-0.024	-1.071	1.030	-1.758	14.66	2.43	26.97	0.9697
	Central-West	-1.738	-2.254	-1.221	-1.738	14.96	2.60	27.32	<.0001

Notes: intercept model before 2001 $\beta_0 =$ -0.583 intercept model after 2001 $\beta_0 =$ -1.7376

[a] $Predict = \beta_0 + \beta_1 Region$ [b] $Estimated\ prevalence = \frac{1}{1 + e^{[-(predict)]}}$

Heterogeneity model before 2001: I^2 = 94.2929%; Test of Moderators: QM (df = 3) = 2.0550, p-value = 0.5611

Heterogeneity model after 2001: I^2 = 84.5612%, Test of Moderators: QM (df = 4) = 28.1287, p-value < 0.0001

NA: No estimates were available, since no study was implemented in that time frame.

Briefly, the meta-regression that considered both geographic region and study year (**Table 2c**) found that studies conducted in the North and Northeast after 2001 had significantly lower HCV prevalence rates than the summary measure (**Figure 1**).

2.1.4. Trim-and-fill

Figure 2 shows the funnel plot and the trim-and-fill analysis. Visual inspection highlighted the relevant role of both publication bias and between-studies heterogeneity (see the main body of the text for additional remarks on the presence/absence of reported information).

There are few dispersion filled-in dots under the lines defining the 95% CIs relative to the potential bias.

The summary measure – adjusted after the inclusion of 11 additional studies by imputation – is 0.2923 [95%CI:0.2136-0.3858] (**Chart 2**). Cochran's Q test showed a p-value=<0.000, corroborating the graphic presentation of the observed biases. Eggers' test also indicates the presence of funnel plot asymmetry (value = -6.415; p-value = 0.009).

Chart 2. Trim-and-fill estimates for HCV infection

```
Number of studies combined: k = 43 (with 11 additional imputed studies)

                          proportion         95%-CI
Random effects model      0.2923   [0.2136; 0.3858]

Test of heterogeneity:
 Q           d.f.   p-value
 2253.70   42      0
```

Figure 2. Funnel plot and trim-and-fill visualization for studies assessing HCV infection.

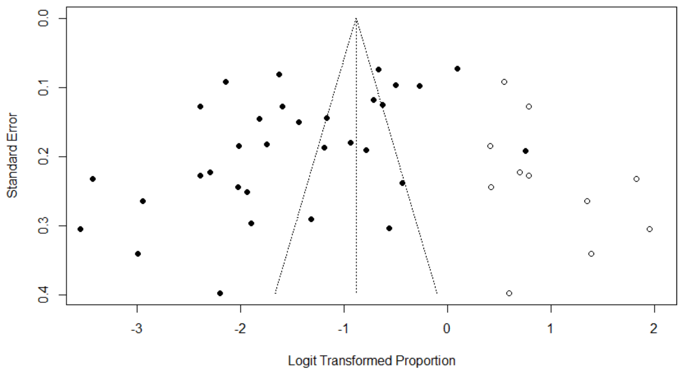

2.1.5. Influence analysis

Figure 3 depicts the Baujat plot for studies estimating HCV infection. The study by Medeiros et al. (2004) was found to have a large influence on both overall heterogeneity and effect. A second study (Silva et al., 2004) was found to have a pronounced influence on overall heterogeneity, but not on overall effect.

Five studies with large samples (compared to the others) had a relevant influence on the meta-analysis summary measure (**Figure 4**). It is possible to view one study with a sample size that is not as large, but with high prevalence, compared to the others.

The influence of publication year on overall prevalence of HCV infection is depicted in **Figure 5**. The oldest study of the whole series had a prevalence of 0.70.

Figure 3. Baujat plot for studies assessing HCV infection.

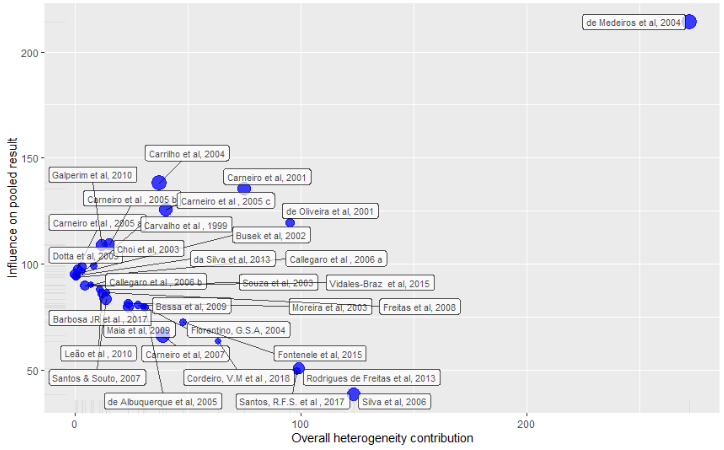

Figure 4. Influence analysis of sample size for studies assessing HCV infection.

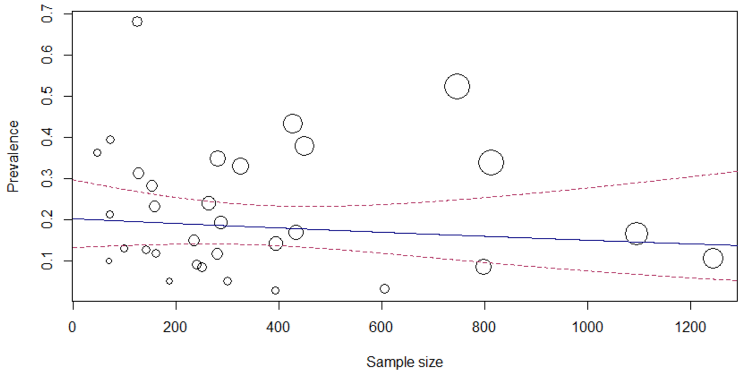

Figure 5. Influence analysis of year of publication for studies assessing HCV infection.

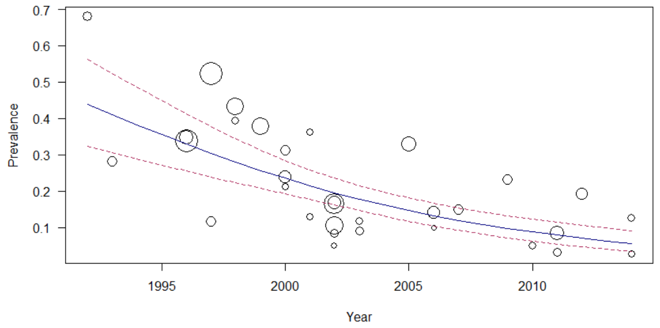

2.2 Prevalence of active HCV infection

2.2.1. Forest plot Q, heterogeneity, and I^2 statistics

Figure 6 displays studies assessing prevalence of active HCV infection. The forest plot helps visualize the heterogeneity described in the main body of the text (the scattered distribution around the vertical axes). Heterogeneity has been assessed by different statistics, as summarized in **Chart 3** as follows:

Figure 6. Forest plot of studies on prevalence of active HCV infection

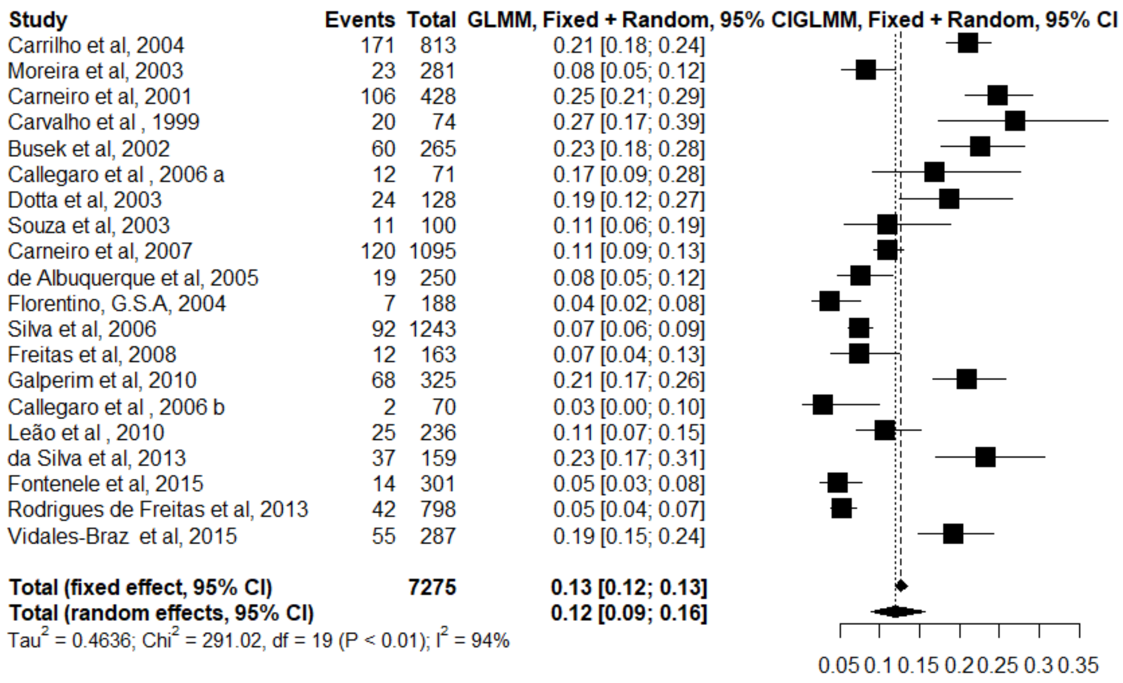

Chart 3. Heterogeneity assessment by different statistics (n=20)

Random effects model = 0.1189; 95%CI [0.0895 - 0.1562]
Chi-square = 291.02; df = 19; p-value: <0.01
I^2 = 98%
Cochran Q Test:

Q	d.f.	p-value	Test
1238.62	31	< 0.0001	Wald-type
1520.60	31	< 0.0001	Likelihood-Ratio

Figure 6.a shows studies assessing prevalence of active HCV infection by the year each study was carried out. The forest plot helps visualize the heterogeneity stratified by year of the study (< 2001 and >= 2001).

Figure 6.a. Forest plot of studies on prevalence of active HCV infection by year

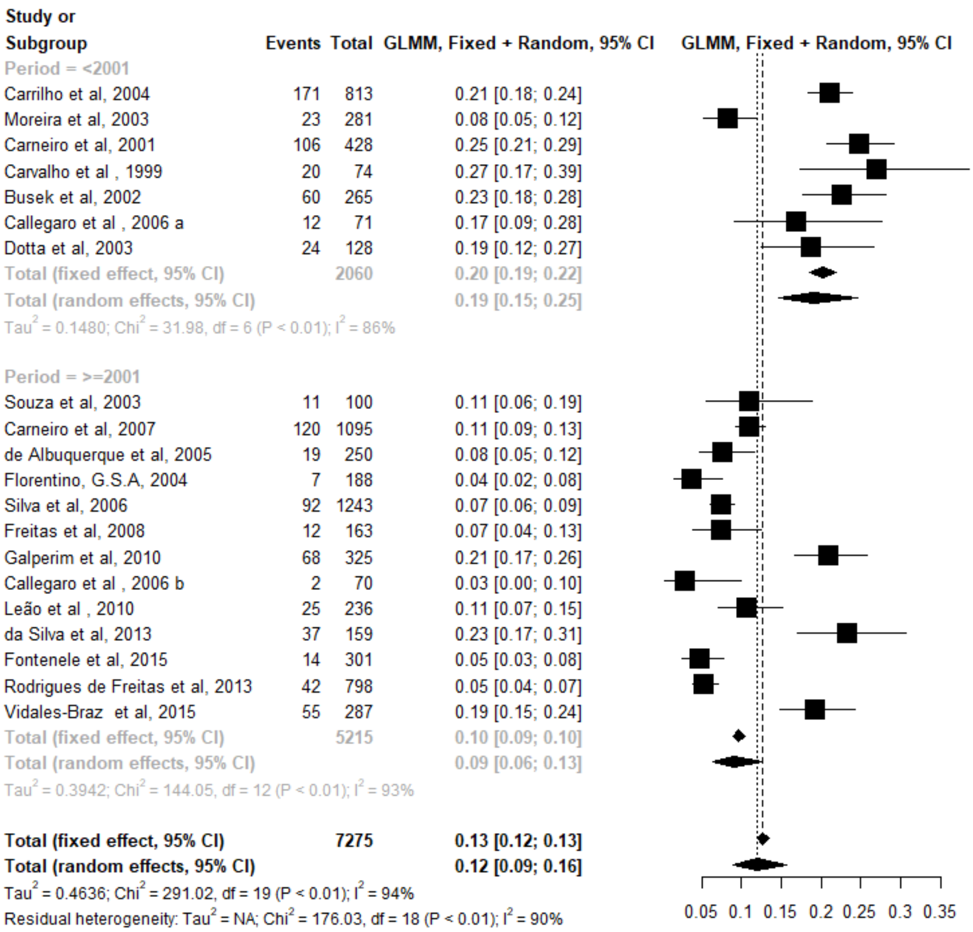

2.2.2. Subgroup analysis

Table 3a summarizes the findings from the subgroup analysis by major geographic regions of Brazil. Summary prevalence rates and respective 95%CIs are stratified by major geographic region of Brazil. Cochran Q test showed p-value <0.001, refuting the null hypothesis (i.e. prevalence did not differ by major geographic region).

Table 3a. Subgroup analysis according to major geographic region of Brazil

Region	K (studies)	Prevalence [95%CI]	tau^2
South	8	0.2019 [0.1845; 0.2204]	0
Southeast	3	0.1281 [0.0751; 0.2100]	0.2368
Central-West	3	0.1318 [0.0718; 0.2297]	0.3213
North	2	0.0672 [0.0388; 0.1139]	0.0730
Northeast	4	0.0627 [0.0464; 0.0843]	0.0218

Test for subgroup differences (random effects model):

	Q	d.f.	p-value
Between groups	75.59	4	0.0001

Table 3b summarize the findings from the subgroup analysis by the year each study was carried out. Summary prevalence rates and respective 95%CIs are stratified by year. Cochran Q Test had a p-value <0.001, refuting the null hypothesis (i.e. prevalence did not differ according to the year the studies were carried out).

Table 3b. Subgroup analysis for the year of the studies

Year	K (studies)	Prevalence [95%CI]	tau^2
< 2001	7	0.1911 [0.1464; 0.2457]	0.1480
>= 2001	13	0.0909 [0.0647; 0.1263]	0.3942

Test for subgroup differences (random effects model):

	Q	d.f.	p-value
Between groups	11.89	1	0.0006

2.2.3. Meta-regression

Table 4a presents the findings from the meta-regression for prevalence of active HCV infection by major geographic region. The North and Northeast regions of Brazil were the main driver of heterogeneity. The differences between the estimates, stratified by major geographic regions, were statistically significant at p-value < 0.000).

Table 4a. Findings from the meta-regression for prevalence of active HCV infection, by major geographic region

Region	Coefficient model			Predict[a]	Estimated prevalence [b]			P-value
	Coefficient	95% CI			Prevalence	95% CI		
		LL	HL			LL	HL	
North	-0.7262	-1.5440	0.0917	-2.5831	7.02	0.00	18.22	0.0818
Northeast	-0.9333	-1.6020	-0.2646	-2.7899	5.79	0.00	16.02	0.0062
South	0.3791	-0.2023	0.9604	-1.4788	18.58	1.53	35.62	0.2012
Southeast	-0.0520	-0.7496	0.6457	-1.9089	12.91	0.00	27.61	0.8839
Central-West	-1.8570	-2.3460	-1.367	-1.8570	13.51	0.00	28.49	<.0001

Notes: intercept model $\beta_0 = $ -1.857 [a] $Predict = \beta_0 + \beta_1 Region$ [b] $Estimated\ prevalence = \frac{1}{1+ e^{[-(predict)]}}$

Heterogeneity: I^2 = 83.0441%; Test of Moderators: QM(df = 4) = 25.2679, p-value = <0.0001

The findings for prevalence of active HCV infection were consistent with those for HCV prevalence. Prevalence of active HCV infection for North and Northeast regions of Brazil were lower than the summary measures using REM as the modeling strategy (Figure 6).

Table 4b shows the findings from the meta-regression for prevalence of active HCV infection by the year each study was carried out. The differences between the estimates, stratified by year, were statistically significant (p-value <0.000).

Table 4b. Findings from the meta-regression for prevalence of active HCV infection, by year

Year	Coefficient model			Predict[a]	P-value
	Coefficient	95% CI			
		LL	HL		
< 2001	-1.4503	-1.8840	-1.0168	-1.4503	< 0.001
>= 2001	-0.8408	-1.3837	-0.2979	-2.2911	0.0024

Notes: intercept model $\beta_0 = $ -1.4503 [a] $Predict = \beta_0 + \beta_1 Year$

Heterogeneity: I^2 = 91.2086%; Test of Moderators: QM (df = 1) = 9.2140, p-value = 0.0024

Consistent with the findings on HCV prevalence, prevalence of active HCV infection was lower compared to summary measures using REM (Figure 6).

Table 4c. Meta-regression for prevalence of active HCV infection by year and major geographic region

Year	Region	Coefficient model			Predict[a]	Estimated prevalence [b]			P-value
		Coefficient	95% CI			Prevalence	95% CI		
			LL	HL			LL	HL	
Before 2001	North	NA	-	-	-	-	-	-	-
	Northeast	NA	-	-	-	-	-		-
	South	-0.232	-0.953	0.490	-1.345	20.66	2.92	38.40	0.5295
	Southeast	-0.666	-1.454	0.123	-1.779	14.43	6.63	29.84	0.0981
	Central-West	-1.114	-1.738	-0.490	-1.114	24.71	5.80	43,61	0.0005
After 2001	North	-0.423	-1.083	0.236	-2,658	6.55	3.88	17.4	0.2084
	Northeast	-0.504	-1.000	-0.008	-2,739	6.07	4.64	16.54	0.0463
	South	0.719	0.233	1.206	-1,515	18.01	1.17	34.86	0.0037
	Southeast	0.092	-0.629	0.814	-2,142	10.51	7.26	23.95	0.2511
	Central-West	-2.235	-2.641	-1.828	-2.235	9.67	8.90	22.62	<0.0001

Notes: intercept model before 2001 $\beta_0 =$ -1.1139 intercept model after 2001 $\beta_0 =$ -2.2346

[a] $Predict = \beta_0 + \beta_1 Region$ [b] $Estimated\ prevalence = \frac{1}{1+ e^{[-(predict)]}}$

Heterogeneity model before 2001: I^2 = 69.4607%; Test of Moderators: QM (df = 2) = 3.2135, p-value = 0.2005

Heterogeneity model after 2001: I^2 =52.0236 %, Test of Moderators: QM (df = 4) = 35.1996, p-value < .0001

NA: No estimates were available, since no study was implemented in that time frame.

As previously observed for HCV prevalence, meta-regression taking into consideration both geographic region and study year highlighted lower prevalence of active HCV infection in the North and Northeast (after 2001) compared to the summary measure estimated by **REM** (**Figure 6**)

2.2.4. Trim-and-fill

Figure 7 shows the funnel plot and trim-and-fill analysis. Visual inspection highlighted the relevant roles of publication bias and between-studies heterogeneity (see the main body of the text for additional remarks on the presence/absence of reported information).

There are few dispersion filled-in dots under the lines defining the 95% CI relative to the potential bias.

The summary measure – adjusted after the inclusion of five additional studies by imputation – is 0.1569 [0.1155; 0.2095] (**Chart 4**). Cochran's Q test had a p-value=<0.000, corroborating the graphic presentation of the observed biases. Eggers' test indicates the presence of funnel plot asymmetry (value = -2.704; p-value = 0.000).

Chart 2. Trim-and-fill estimates for hepatitis C

Number of studies combined: k = 25 (with 5 added studies)

	proportion	95%-CI
Random effects model	0.1569	[0.1155; 0.2095]

Test of heterogeneity:

Q	d.f.	p-value
418.69	24	< 0.0001

Figure 7. Funnel plot and trim-and-fill visualization for studies assessing the prevalence of active HCV infection

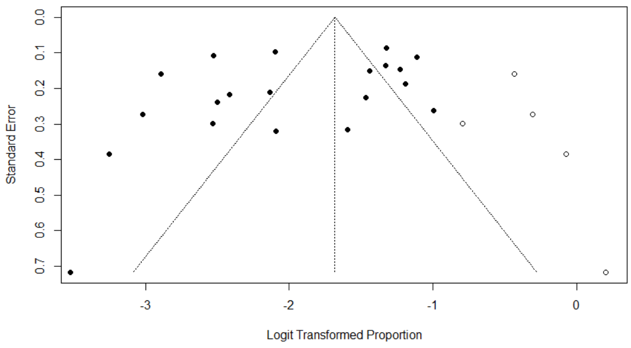

2.2.5. Influence analysis

Figure 8 displays the Baujat plot for studies estimating hepatitis C. The studies by Carrilho et al. (2004), Carneiro et al. (2001), Rodrigues e Freitas et al. (2013), and Silva et al. (2006) were found to have a large influence on both overall heterogeneity and effect.

Four studies with large samples, compared to the others, had a relevant influence on meta-analysis summary measure (**Figure 9**). It is possible to see one study with a sample size that is not as large, but with high prevalence, compared to the others.

Figure 10 shows the influence of publication year on overall prevalence of active HCV infection. There is a dispersion of the studies under analysis. However, there is no discernible pattern that might document the influence of year of publication on prevalence of active HCV infection meta-analysis.

Figure 8. Baujat Plot for studies assessing prevalence of active HCV infection

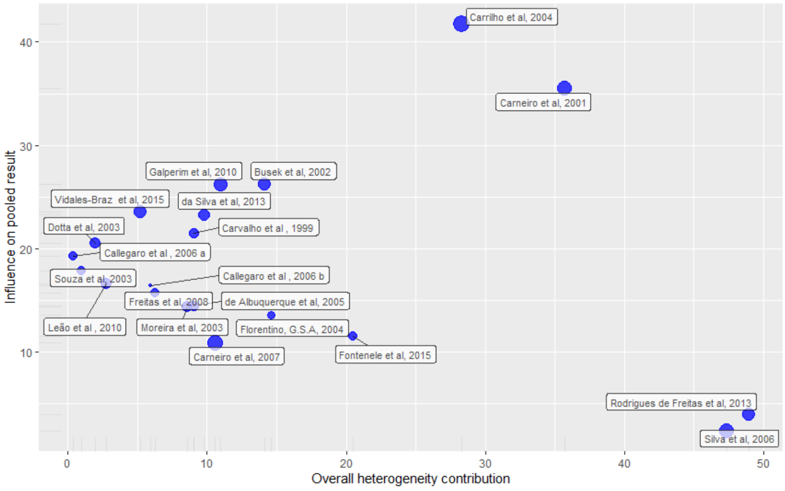

Figure 9. Influence analysis of sample sizes for studies assessing prevalence of active HCV infection

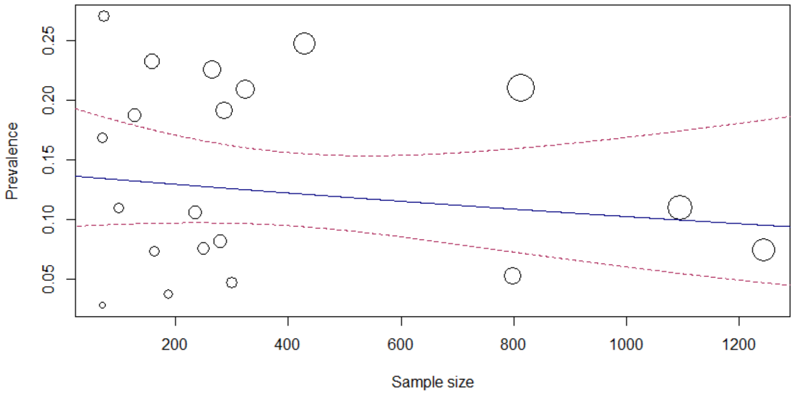

Figure 10. Influence analysis of year of publication for studies assessing prevalence of active HCV infection

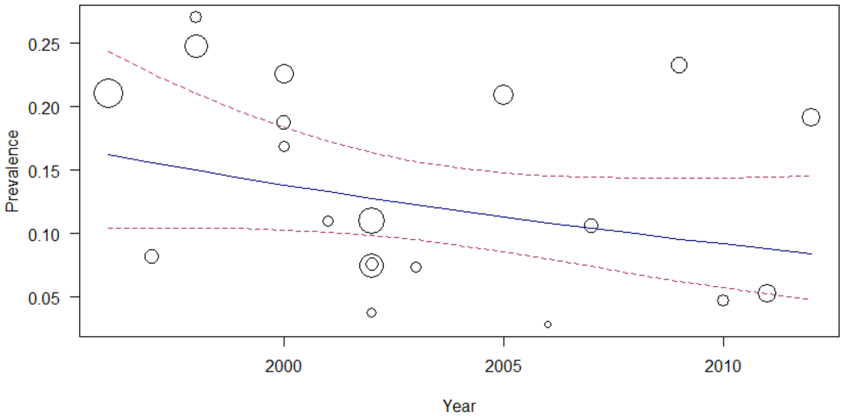

References:

An C, O'Malley AJ, Rockmore DN. Referral paths in the U.S. physician network. Appl Netw Sci. 2018;3(1):20. doi: 10.1007/s41109-018-0081-4.

Baujat B, Mahé C, Pignon JP, Hill C. A graphical method for exploring heterogeneity in meta-analyses: application to a meta-analysis of 65 trials. Stat Med. 2002;21(18):2641-52. doi: 10.1002/sim.1221.

Borenstein, M., Hedges, L. V., Higgins, J. P. T., & Rothstein, H. *Introduction to Meta-analysis*. Chichester, U.K: John Wiley & Sons, 2009.

Duval, S. J. The trim and fill method. In: H. R. Rothstein, A. J. Sutton, & M. Borenstein (Eds.). *Publication Bias in Meta-analysis: Prevention, assessment, and adjustments* (pp. 127-144). Chichester, U.K.: John Wiley & Sons, 2005.

Egger M, Davey Smith G, Schneider M, Minder C. Bias in meta-analysis detected by a simple, graphical test. BMJ. 1997;315(7109):629-34. doi: 10.1136/bmj.315.7109.629.

Harrer M, Cuijpers P, Furukawa TA, Ebert DD. *Doing Meta-Analysis in R: A Hands-on Guide,* 2019. E-book available at: https://bookdown.org/MathiasHarrer/Doing_Meta_Analysis_in_R/. DOI: 10.5281/zenodo.2551803.

Higgins JP, Thompson SG, Deeks JJ, Altman DG. Measuring inconsistency in meta-analyses. BMJ. 2003;327(7414):557-60. doi: 10.1136/bmj.327.7414.557. .

Hoaglin DC. Misunderstandings about Q and 'Cochran's Q test' in meta-analysis. Stat Med. 2016;35(4):485-95. doi: 10.1002/sim.6632.

Oxman AD, Guyatt GH. A consumer's guide to subgroup analyses. Ann Intern Med. 1992;116(1):78-84. doi: 10.7326/0003-4819-116-1-78.

Viechtbauer W, Cheung MW. Outlier and influence diagnostics for meta-analysis. Res Synth Methods. 2010;1(2):112-25. doi: 10.1002/jrsm.11.

Yusuf S, Wittes J, Probstfield J, Tyroler HA. Analysis and interpretation of treatment effects in subgroups of patients in randomized clinical trials. JAMA. 1991;266(1):93-8.
