# Supplementary material for: Persistently high hepatitis C rates in haemodialysis patients in Brazil [a systematic review and meta-analysis]
Source: Sci Rep. 2022 Jan 10;12:330. doi: 10.1038/s41598-021-03961-x (PMC8748660; doi:10.1038/s41598-021-03961-x)
Supplement: Supplementary file 4 — Supplementary Information 4. [file 41598_2021_3961_MOESM4_ESM.pdf]

Table S4. Summary of the quality appraisal for the included studies using JBI (Joanna Briggs Institute) critical appraisal checklist for prevalence studies

Author	1. Was the sample frame appropriate to address the target population?	2. Were study participants sampled in an appropriate way?	3. Was the sample size adequate?	4. Were the study subjects and the setting described in detail?	5. Was the data analysis conducted with sufficient coverage of the identified sample?	6. Were valid methods used for the identification of the condition?	7. Was the condition measured in a standard, reliable way for all participants?	8. Was there appropriate statistical analysis?	9. Was the response rate adequate, and if not, was the low response rate managed appropriately?	Score
Bessa et al, 2009	yes	unclear	unclear	yes	yes	yes	yes	yes	unclear	6
Busek et al, 2002	no	no	unclear	yes	yes	yes	yes	yes	unclear	5
Carneiro et al, 2001	yes	yes	yes	no	yes	yes	yes	yes	yes	8
Carneiro et al, 2007	yes	yes	yes	no	yes	yes	yes	yes	yes	8
Carrilho et al, 2004	yes	unclear	unclear	yes	yes	yes	yes	yes	unclear	6
Choi et al, 2003	yes	yes	yes	yes	unclear	yes	yes	no	yes	7
da Silva et al, 2013	yes	unclear	unclear	yes	yes	yes	yes	yes	unclear	6
De Albuquerque et al, 2005	yes	yes	yes	no	yes	yes	yes	yes	yes	8
de Medeiros et al, 2004	yes	yes	yes	yes	yes	yes	yes	yes	yes	9
de Oliveira et al, 2001	yes	unclear	unclear	no	yes	yes	yes	no	unclear	4
Dotta et al, 2003	yes	unclear	unclear	yes	yes	yes	yes	yes	unclear	6
Fontenele et al, 2015	yes	yes	yes	yes	yes	yes	yes	yes	unclear	8
Freitas et al, 2008	yes	yes	yes	yes	yes	yes	yes	yes	unclear	8
Galperim et al, 2010	yes	yes	yes	yes	yes	yes	yes	yes	unclear	8
Maia et al, 2009	yes	yes	yes	yes	yes	yes	yes	yes	unclear	8
Moreira et al, 2003	yes	yes	yes	no	yes	yes	yes	no	unclear	6
Rodrigues de Freitas et al, 2013	yes	yes	yes	yes	yes	yes	yes	yes	yes	9
Santos & Souto, 2007	yes	yes	yes	yes	yes	yes	yes	no	unclear	7
Silva et al, 2006	yes	unclear	unclear	no	yes	yes	yes	yes	yes	6
Souza et al, 2003	yes	yes	yes	no	yes	yes	yes	yes	yes	8
Vidales-Braz et al, 2015	yes	yes	yes	yes	yes	yes	yes	yes	yes	9

Author	1. Was the sample frame appropriate to address the target population?	2. Were study participants sampled in an appropriate way?	3. Was the sample size adequate?	4. Were the study subjects and the setting described in detail?	5. Was the data analysis conducted with sufficient coverage of the identified sample?	6. Were valid methods used for the identification of the condition?	7. Was the condition measured in a standard, reliable way for all participants?	8. Was there appropriate statistical analysis?	9. Was the response rate adequate, and if not, was the low response rate managed appropriately?	Score
Carvalho et al, 1999	yes	yes	yes	unclear	unclear	yes	yes	yes	unclear	6
Leão et al, 2010	yes	yes	yes	yes	yes	yes	yes	yes	yes	9
Cordeiro, V.M et al, 2018	yes	yes	yes	yes	yes	yes	yes	yes	yes	9
Santos, R.F.S. et al, 2017	yes	yes	unclear	yes	yes	yes	yes	yes	yes	8
Barbosa JR et al, 2017	yes	yes	yes	yes	yes	yes	yes	yes	unclear	8
Florentino, G.S.A, 2004	yes	yes	unclear	yes	yes	yes	yes	yes	yes	8
Callegaro et al, 2006	yes	unclear	unclear	yes	no	yes	yes	no	unclear	4
Carneiro et al, 2006b	yes	unclear	unclear	yes	yes	yes	yes	yes	yes	7

JBI criteria can be found as follows: https://jbi.global/critical-appraisal-tools
