# Supplementary material for: Persistently high hepatitis C rates in haemodialysis patients in Brazil [a systematic review and meta-analysis]
Source: Sci Rep. 2022 Jan 10;12:330. doi: 10.1038/s41598-021-03961-x (PMC8748660; doi:10.1038/s41598-021-03961-x)
Supplement: Supplementary file 5 — Supplementary Information 5. [file 41598_2021_3961_MOESM5_ESM.pdf]

Table 5S: Tests approved by Agência de Vigilância Sanitária (ANVISA, Brazilian Health Regulatory Agency) valid for the period 2012-2030

**Molecular tests**					
**Product**	**Technical name**	**Company**	**Manufacturer**	**Registration**	**Expiration date**
ABBOTT REAL TIME HCV AMPLIFICATION REAGENT KIT / ABBOTT REALTIME HCV KIT REAGENTE DE AMPLIFICAÇÃO	MONITORAMENTO DE CARGA VIRAL – VÍRUS DE HEPATITE C (HCV)	ABBOTT LABORATÓRIOS DO BRASIL LTDA	ABBOTT MOLECULAR INC.	80146501585	5/13/2024
ABBOTT REALTIME HCV CONTROLS - ABBOTT REALTIME HCV CONTROLES	MONITORAMENTO DE CARGA VIRAL – VÍRUS DE HEPATITE C (HCV)	ABBOTT LABORATÓRIOS DO BRASIL LTDA	ABBOTT MOLECULAR INC.	80146501586	5/13/2024
ABBOTT REALTIME HCV CALIBRATORS / ABBOTT REALTIME HCV CALIBRADORES	MONITORAMENTO DE CARGA VIRAL – VÍRUS DE HEPATITE C (HCV)	ABBOTT LABORATÓRIOS DO BRASIL LTDA	ABBOTT MOLECULAR INC.	80146501587	5/13/2024
Abbott RealTime HCV Genotype II Controls / Abbott RealTime HCV Genótipo II Controles	ÁCIDO NUCLÉICO DE VÍRUS DE HEPATITE C (HCV)	ABBOTT LABORATÓRIOS DO BRASIL LTDA	ABBOTT MOLECULAR INC.	80146501755	4/25/2026
Abbott RealTime HCV Genotype II Amplification Reagent Kit / Abbott RealTime HCV Genótipo II Kit Reagente de Amplificação	ÁCIDO NUCLÉICO DE VÍRUS DE HEPATITE C (HCV)	ABBOTT LABORATÓRIOS DO BRASIL LTDA	ABBOTT MOLECULAR INC.	80146501756	4/25/2026
Família Alinity m HCV	ÁCIDO NUCLÉICO DE VÍRUS DE HEPATITE C (HCV)	ABBOTT LABORATÓRIOS DO BRASIL LTDA	ABBOTT MOLECULAR INC.	80146502222	9/2/2029
DETECT TM HCV	Detec. ou Quantific. Antig. Anticorpos Hepatite C	Alka tecnologia em diagnostico com. imp. exp. de produtos ltda	ADALTIS INC	10269670003	3/19/2011
Xpert HCV Viral Load	MONITORAMENTO DE CARGA VIRAL – VÍRUS DE HEPATITE C (HCV)	Cepheid Brasil Importação, Exportação e Comércio de Produtos de Diagnósticos ltda	CEPHEID AB	81062710024	7/31/2027
HCV RNA QUANTITATIVO PARA PCR EM TEMPO REAL	MONITORAMENTO DE CARGA VIRAL – VÍRUS DE HEPATITE C (HCV)	NUCLEAR LASER MEDICINE, COMÉRCIO, IMPORTAÇÃO E EXPORTAÇÃO DE KITS	NUCLEAR LASER MEDICINE S.R.L	80578810006	9/26/2011

		ELETROMEDICINAIS E DIAGNÓSTICOS LTDA			
HCV RNA QUALITATIVO PARA PCR EM TEMPO REAL	ÁCIDO NUCLÉICO DE VÍRUS DE HEPATITE C (HCV)	NUCLEAR LASER MEDICINE, COMÉRCIO, IMPORTAÇÃO E EXPORTAÇÃO DE KITS ELETROMEDICINAIS E DIAGNÓSTICOS LTDA	NUCLEAR LASER MEDICINE S.R.L	80578810009	1/4/2016
Família Aptima® HCV Quant Dx Assay	ÁCIDO NUCLÉICO DE VÍRUS DE HEPATITE C (HCV)	PASSROD IMPORTAÇÃO E EXPORTAÇÃO DE PRODUTOS PARA SAÚDE LTDA - ME	HOLOGIC, INC	81504790045	12/24/2028
artus HCV RG RT- PCR Kit	MONITORAMENTO DE CARGA VIRAL – VÍRUS DE HEPATITE C (HCV)	QIAGEN BIOTECNOLOGIA BRASIL LTDA.	QIAGEN GMBH	10322250037	1/11/2025
artus HCV QS-RGQ	MONITORAMENTO DE CARGA VIRAL – VÍRUS DE HEPATITE C (HCV)	QIAGEN BIOTECNOLOGIA BRASIL LTDA.	QIAGEN GMBH	10322250060	9/28/2025
Reagente de Sonda Discriminatória HCV PROCLEIX	ÁCIDO NUCLÉICO DE VÍRUS DE HEPATITE C (HCV)	REM INDUSTRIA E COMERCIO LTDA	GEN-PROBE INCORPORATED	80144950535	3/23/2025
AMPLICOR HCV SPECIMEN PREPARATION KIT V2.0	ÁCIDO NUCLÉICO DE VÍRUS DE HEPATITE C (HCV)	ROCHE DIAGNÓSTICA BRASIL LTDA	ROCHE MOLECULAR SYSTEMS	10287410526	8/18/2025
AMPLICOR HCV AMPLIFICATION KIT V2.0	ÁCIDO NUCLÉICO DE VÍRUS DE HEPATITE C (HCV)	ROCHE DIAGNÓSTICA BRASIL LTDA	ROCHE MOLECULAR SYSTEMS	10287410527	8/18/2025
AMPLICOR HCV CONTROLS KIT, V 2.0	MONITORAMENTO DE CARGA VIRAL – VÍRUS DE HEPATITE C (HCV)	ROCHE DIAGNÓSTICA BRASIL LTDA	ROCHE MOLECULAR SYSTEMS	10287410537	9/12/2025
COBAS AMPLICOR HCV DETECTION KIT, V 2.0	ÁCIDO NUCLÉICO DE VÍRUS DE HEPATITE C (HCV)	ROCHE DIAGNÓSTICA BRASIL LTDA	ROCHE MOLECULAR SYSTEMS	10287410554	4/3/2016
AMPLICOR HCV MONITOR TEST, VERSÃO 2.0	ÁCIDO NUCLÉICO DE VÍRUS DE HEPATITE C (HCV)	ROCHE DIAGNÓSTICA BRASIL LTDA	ROCHE MOLECULAR SYSTEMS	10287410639	7/16/2012

LINEAR ARRAY DETECTION KIT	ÁCIDO NUCLÉICO DE VÍRUS DE HEPATITE C (HCV)	ROCHE DIAGNÓSTICA BRASIL LTDA	ROCHE MOLECULAR SYSTEMS	10287410640	7/16/2017
COBAS AMPLICOR HCV MONITOR TEST, VERSÃO 2.0	ÁCIDO NUCLÉICO DE VÍRUS DE HEPATITE C (HCV)	ROCHE DIAGNÓSTICA BRASIL LTDA	ROCHE MOLECULAR SYSTEMS	10287410641	7/16/2012
COBAS AMPLIPREP/COBAS TAQMAN HCV	ÁCIDO NUCLÉICO DE VÍRUS DE HEPATITE C (HCV)	ROCHE DIAGNÓSTICA BRASIL LTDA	ROCHE MOLECULAR SYSTEMS	10287410694	4/22/2013
HCV GENOTYPING TEST	ANTÍGENO DE VÍRUS DE HEPATITE C (HCV)	ROCHE DIAGNÓSTICA BRASIL LTDA	ROCHE MOLECULAR SYSTEMS	10287410743	1/8/2018
COBAS TAQMAN HCV TEST	MONITORAMENTO DE CARGA VIRAL – VÍRUS DE HEPATITE C (HCV)	ROCHE DIAGNÓSTICA BRASIL LTDA	ROCHE MOLECULAR SYSTEMS	10287410746	7/21/2023
High Pure System Viral Nucleic Acid Kit	ÁCIDO NUCLÉICO DE VÍRUS DE HEPATITE C (HCV)	ROCHE DIAGNÓSTICA BRASIL LTDA	ROCHE MOLECULAR SYSTEMS	10287410778	2/16/2024
COBAS® AmpliPrep/COBAS® TaqMan® HCV Qualitative Test, v2.0	ÁCIDO NUCLÉICO DE VÍRUS DE HEPATITE C (HCV)	ROCHE DIAGNÓSTICA BRASIL LTDA	ROCHE MOLECULAR SYSTEMS	10287410994	2/13/2028
COBAS® AmpliPrep/COBAS® TaqMan® HCV Quantitative Test, v2.0	MONITORAMENTO DE CARGA VIRAL – VÍRUS DE HEPATITE C (HCV)	ROCHE DIAGNÓSTICA BRASIL LTDA	ROCHE MOLECULAR SYSTEMS	10287410995	3/4/2028
COBAS AMPLIPREP/COBAS TAQMAN HCV	MONITORAMENTO DE CARGA VIRAL – VÍRUS DE HEPATITE C (HCV)	ROCHE DIAGNÓSTICA BRASIL LTDA	ROCHE MOLECULAR SYSTEMS	10287411084	3/23/2025
COBAS HCV GT CONTROL KIT	ÁCIDO NUCLÉICO DE VÍRUS DE HEPATITE C (HCV)	ROCHE DIAGNÓSTICA BRASIL LTDA	ROCHE DIAGNOSTICS GMBH	10287411149	8/1/2026

COBAS HCV	ÁCIDO NUCLÉICO DE VÍRUS DE HEPATITE C (HCV)	ROCHE DIAGNÓSTICA BRASIL LTDA	ROCHE DIAGNOSTICS GMBH	10287411152	8/1/2026
COBAS HCV	ÁCIDO NUCLÉICO DE VÍRUS DE HEPATITE C (HCV)	ROCHE DIAGNÓSTICA BRASIL LTDA	ROCHE DIAGNOSTICS GMBH	10287411181	12/19/2026
VERSANT HCV RNA 3.0 (BDNA)	MONITORAMENTO DE CARGA VIRAL – VÍRUS DE HEPATITE C (HCV)	SIEMENS HEALTHCARE DIAGNÓSTICOS LTDA	SIEMENS HEALTHCARE DIAGNOSTICS INC	10345160642	11/27/2017
VERSANT HCV RNA QUALITATIVO	ANTÍGENO DE VÍRUS DE HEPATITE C (HCV)	SIEMENS HEALTHCARE DIAGNÓSTICOS LTDA	SIEMENS HEALTHCARE DIAGNOSTICS INC	10345160844	8/11/2013
VERSANT HCV RNA QUALITATIVE ASSAY CONTROL SET	ANTÍGENO DE VÍRUS DE HEPATITE C (HCV)	SIEMENS HEALTHCARE DIAGNÓSTICOS LTDA	SIEMENS HEALTHCARE DIAGNOSTICS INC	10345160846	8/11/2013
VERSANT HCV AMPLIFICATION 2.0 KIT (LIPA)	MONITORAMENTO DE CARGA VIRAL – VÍRUS DE HEPATITE C (HCV)	SIEMENS HEALTHCARE DIAGNÓSTICOS LTDA	INNOGENETICS N.V. - BÉLGICA PARA SIEMENS HEALTHCARE DIAGNOSTICS, INC.	10345160847	8/11/2023
VERSANT HCV GENOTYPE 2.0 ASSAY (LIPA)	ANTÍGENO DE VÍRUS DE HEPATITE C (HCV)	SIEMENS HEALTHCARE DIAGNÓSTICOS LTDA	INNOGENETICS N.V. - BÉLGICA PARA SIEMENS HEALTHCARE DIAGNOSTICS, INC.	10345160848	8/11/2023
VERSANT HCV CONTROL 2.0 KIT (LIPA)	ANTÍGENO DE VÍRUS DE HEPATITE C (HCV)	SIEMENS HEALTHCARE DIAGNÓSTICOS LTDA	INNOGENETICS N.V. - BÉLGICA PARA SIEMENS HEALTHCARE DIAGNOSTICS, INC.	10345160849	8/11/2023
COBAS HCV GT	ANTÍGENO DE VÍRUS DE HEPATITE C (HCV)	ROCHE DIAGNÓSTICA BRASIL LTDA	ROCHE DIAGNOSTICS GMBH	10287411151	8/1/2026
VERSANT AUTO DETECT SET	ANTÍGENO DE VÍRUS DE HEPATITE C (HCV)	SIEMENS HEALTHCARE DIAGNÓSTICOS LTDA	SIEMENS HEALTHCARE DIAGNOSITCS INC	10345160843	8/11/2013
**Serological tests**					
**Product**	**Technical name**	**Company**	**Manufacturer**	**Registration**	**Registration expiration**
IMMUNOCOMB II HCV	ANTICORPO TOTAL PARA VÍRUS DE HEPATITE C (ANTI-HCV)	ABBOTT DIAGNOSTICOS RAPIDOS S.A.	ORGENICS LTD	10071770663	7/29/2019

Bioline HCV	ANTICORPO TOTAL PARA VÍRUS DE HEPATITE C (ANTI-HCV)	ABBOTT DIAGNOSTICOS RAPIDOS S.A.	ABBOT DIAGNOSTICS KOREA. INC	10071770685	9/4/2026
HCV ELISA TEST BIOEASY	ANTICORPO TOTAL PARA VÍRUS DE HEPATITE C (ANTI-HCV)	ABBOTT DIAGNOSTICOS RAPIDOS S.A.	ACON BIOTECH CO. LTD	10071770702	6/24/2019
HCV 3.0 ELISA	ANTICORPO TOTAL PARA VÍRUS DE HEPATITE C (ANTI-HCV)	ABBOTT DIAGNOSTICOS RAPIDOS S.A.	STANDARD DIAGNOSTIC, INC	10071770767	6/21/2021
HCV Hepatitis C Virus Rapid Test Device	ANTICORPO TOTAL PARA VÍRUS DE HEPATITE C (ANTI-HCV)	ABBOTT DIAGNOSTICOS RAPIDOS S.A.	ABON BIOPHARM	10071770813	5/31/2026
PRISM HCV (KIT DE ENSAIO)	ANTICORPO TOTAL PARA VÍRUS DE HEPATITE C (ANTI-HCV)	ABBOTT LABORATÓRIOS DO BRASIL LTDA	ABBOTT GMBH & CO. KG	10055310639	8/9/2021
AXSYM HCV 3.0 CONTROLES	ANTICORPO TOTAL PARA VÍRUS DE HEPATITE C (ANTI-HCV)	ABBOTT LABORATÓRIOS DO BRASIL LTDA	ABBOTT GMBH & CO. KG	10055310682	5/30/2016
AxSYM HCV 3.0 REAGENTES	ANTICORPO TOTAL PARA VÍRUS DE HEPATITE C (ANTI-HCV)	ABBOTT LABORATÓRIOS DO BRASIL LTDA	ABBOTT GMBH & CO. KG	10055310685	9/5/2016
IMX HCV 3.0 REAGENTES	ANTICORPO TOTAL PARA VÍRUS DE HEPATITE C (ANTI-HCV)	ABBOTT LABORATÓRIOS DO BRASIL LTDA	ABBOTT GMBH & CO. KG	10055310741	3/12/2013
IMX HCV 3.0 CONTROLES	ANTICORPO TOTAL PARA VÍRUS DE HEPATITE C (ANTI-HCV)	ABBOTT LABORATÓRIOS DO BRASIL LTDA	ABBOTT GMBH & CO. KG	10055310744	3/12/2013
MUREX ANTI-HCV (VERSION 4.0)	ANTÍGENO DE VÍRUS DE HEPATITE C (HCV)	ABBOTT LABORATÓRIOS DO BRASIL LTDA	MUREX BIOTECH	10055310825	3/30/2014
MUREX HCV SEROTYPING 1-6 ASSAY	Detec.ou Quantific.Antig.Anticorpos Hepatite C	ABBOTT LABORATÓRIOS DO BRASIL LTDA	MUREX BIOTECH LTDA	10055310890	8/11/2004
ARCHITECT Anti-HCV Calibrators	ANTICORPO TOTAL PARA VÍRUS DE HEPATITE C (ANTI-HCV)	ABBOTT LABORATÓRIOS DO BRASIL LTDA	ABBOTT GMBH & CO. KG	10055311123	2/5/2023
ARCHITECT Anti-HCV Controls	ANTICORPO TOTAL PARA VÍRUS DE HEPATITE C (ANTI-HCV)	ABBOTT LABORATÓRIOS DO BRASIL LTDA	ABBOTT GMBH & CO. KG	10055311124	2/5/2023
ARCHITECT Anti-HCV Reagents	ANTICORPO TOTAL PARA VÍRUS DE HEPATITE C (ANTI-HCV)	ABBOTT LABORATÓRIOS DO BRASIL LTDA	ABBOTT GMBH & CO. KG	10055311127	3/28/2023
MUREX HCV Ag/Ab COMBINATION / MUREX HCV COMBINAÇÃO Ag/Ab	Detec.ou Quantific.Antig.Anticorpos Hepatite C	ABBOTT LABORATÓRIOS DO BRASIL LTDA	MUREX BIOTECH	80146501415	3/12/2012

ARCHITECT HBsAg CONFIRMATORY V.1 CALIBRATORS / ARCHITECT HBsAg CONFIRMATÓRIO V.1 CALIBRADORES	ANTICORPO TOTAL PARA VÍRUS DE HEPATITE C (ANTI-HCV)	ABBOTT LABORATÓRIOS DO BRASIL LTDA	ABBOTT IRELAND	80146501515	5/19/2013
Murex HCV Ag/Ab Combination	ANTÍGENO E ANTICORPO PARA VÍRUS DE HEPATITE C (HCV AG/ANTI-HCV)	ABBOTT LABORATÓRIOS DO BRASIL LTDA	MUREX BIOTECH	80146501713	10/4/2015
ARCHITECT HCV Ag Reagent Kit	ANTÍGENO DE VÍRUS DE HEPATITE C (HCV)	ABBOTT LABORATÓRIOS DO BRASIL LTDA	ABBOTT GMBH & CO.KG.	80146501842	7/8/2023
ARCHITECT HCV Ag Calibrators	ANTÍGENO DE VÍRUS DE HEPATITE C (HCV)	ABBOTT LABORATÓRIOS DO BRASIL LTDA	ABBOTT GMBH & CO.KG.	80146501843	7/8/2023
ARCHITECT HCV Ag Controls	ANTÍGENO DE VÍRUS DE HEPATITE C (HCV)	ABBOTT LABORATÓRIOS DO BRASIL LTDA	ABBOTT GMBH & CO.KG.	80146501844	7/8/2023
Família ARCHITECT Anti-HCV	ANTICORPO TOTAL PARA VÍRUS DE HEPATITE C (ANTI-HCV)	ABBOTT LABORATÓRIOS DO BRASIL LTDA	ABBOTT GMBH	80146502047	11/27/2027
Família Alinity i Anti-HCV	ANTICORPO TOTAL PARA VÍRUS DE HEPATITE C (ANTI-HCV)	ABBOTT LABORATÓRIOS DO BRASIL LTDA	ABBOTT GMBH	80146502051	11/27/2027
Família ARCHITECT HCV Ag	ANTÍGENO DE VÍRUS DE HEPATITE C (HCV)	ABBOTT LABORATÓRIOS DO BRASIL LTDA	ABBOTT GMBH	80146502093	3/26/2028
Família Alinity s Anti-HCV	ANTICORPO TOTAL PARA VÍRUS DE HEPATITE C (ANTI-HCV)	ABBOTT LABORATÓRIOS DO BRASIL LTDA	ABBOTT GMBH	80146502123	5/21/2028
ACCESS HCV AB PLUS QC	ANTICORPO TOTAL PARA VÍRUS DE HEPATITE C (ANTI-HCV)	BIO-RAD LABORATORIOS BRASIL LTDA	BIO-RAD	80020690182	5/19/2028
ACCESS HCV AB PLUS CALIBRADORES	ANTICORPO TOTAL PARA VÍRUS DE HEPATITE C (ANTI-HCV)	BIO-RAD LABORATORIOS BRASIL LTDA	BIO-RAD	80020690183	5/19/2028
ACCESS HCV AB PLUS	ANTICORPO TOTAL PARA VÍRUS DE HEPATITE C (ANTI-HCV)	BIO-RAD LABORATORIOS BRASIL LTDA	BIO-RAD	80020690184	5/19/2028
MONOLISA Anti-HCV PLUS Version 2	ANTICORPO TOTAL PARA VÍRUS DE HEPATITE C (ANTI-HCV)	BIO-RAD LABORATORIOS BRASIL LTDA	BIO-RAD	80020690268	9/5/2016
Monolisa HCV Ag-Ab ULTRA V2	ANTÍGENO E ANTICORPO PARA VÍRUS DE HEPATITE C (HCV AG/ANTI-HCV)	BIO-RAD LABORATORIOS BRASIL LTDA	BIO-RAD	80020690319	6/2/2024

Monolisa Anti-HCV PLUS Version 3	ANTICORPO TOTAL PARA VÍRUS DE HEPATITE C (ANTI-HCV)	BIO-RAD LABORATORIOS BRASIL LTDA	BIO-RAD	80020690348	5/18/2025
Família Access HCV Ab V3	ANTICORPO TOTAL PARA VÍRUS DE HEPATITE C (ANTI-HCV)	BIO-RAD LABORATORIOS BRASIL LTDA	BIO-RAD	80020690380	7/17/2027
Família Geenius HCV Supplemental	ANTICORPO CONFIRMATÓRIO PARA VÍRUS DE HEPATITE C (ANTI-HCV)	BIO-RAD LABORATORIOS BRASIL LTDA	BIO-RAD	80020690405	10/14/2029
HEPANOSTIKA HCV ULTRA	ANTICORPO TOTAL PARA VÍRUS DE HEPATITE C (ANTI-HCV)	BIOMERIEUX BRASIL INDUSTRIA E COMERCIO DE PRODUTOS LABORATORIAIS LTDA	BIOMERIEUX SA	10158120525	9/22/2014
VIDAS ANTI-HCV (HCV)	ANTICORPO TOTAL PARA VÍRUS DE HEPATITE C (ANTI-HCV)	BIOMERIEUX BRASIL INDUSTRIA E COMERCIO DE PRODUTOS LABORATORIAIS LTDA	BIOMERIEUX SA	10158120669	3/4/2028
VIKIA anti-HCV	ANTICORPO TOTAL PARA VÍRUS DE HEPATITE C (ANTI-HCV)	BIOMERIEUX BRASIL INDUSTRIA E COMERCIO DE PRODUTOS LABORATORIAIS LTDA	BIOMERIEUX SA	10158120717	6/24/2019
DETECT PARA HCV	Detec.ou Quantific.Antig.Anticorpos Hepatite C	CAPRICORN TECHNOLOGIES DO BRASIL LTDA	ADALTIS INC	10387650043	11/21/2011
EIAGEN HCV AB KIT	ANTICORPO TOTAL PARA VÍRUS DE HEPATITE C (ANTI-HCV)	CAPRICORN TECHNOLOGIES DO BRASIL LTDA	ADALTIS S.R.L.	10387650106	11/26/2012
HCV MICRO EIA	ANTICORPO TOTAL PARA VÍRUS DE HEPATITE C (ANTI-HCV)	CAPRICORN TECHNOLOGIES DO BRASIL LTDA	MBS MEDICAL BIOLOGICAL SERVICE SRL	10387650109	12/10/2012
SP-NANBASE C-96 3.0 (ANTI-HCV 3ª GERACAO)	ANTICORPO TOTAL PARA VÍRUS DE HEPATITE C (ANTI-HCV)	CAPRICORN TECHNOLOGIES DO BRASIL LTDA	GENERAL BIOLOGICAL CORP.	10387650112	2/11/2013
Teste Rápido OL HCV	ANTICORPO TOTAL PARA VÍRUS DE HEPATITE C (ANTI-HCV)	CHEMBIO DIAGNOSTICS BRAZIL LTDA.	CHEMBIO DIAGNOSTICS BRAZIL LTDA.	80535240022	7/29/2023
OL Check HCV	ANTICORPO TOTAL PARA VÍRUS DE HEPATITE C (ANTI-HCV)	CHEMBIO DIAGNOSTICS BRAZIL LTDA.	CHEMBIO DIAGNOSTICS BRAZIL LTDA.	80535240042	5/11/2025
TESTE RÁPIDO STANDARD Q HCV Ab	ANTICORPO TOTAL PARA VÍRUS DE HEPATITE C (ANTI-HCV)	DESCARPACK DESCARTAVEIS DO BRASIL LTDA	SD BIOSENSOR, INC	10330660252	6/3/2029
HCV Rapid Test	ANTICORPO TOTAL PARA VÍRUS DE HEPATITE C (ANTI-HCV)	DIAGNÓSTICA INDÚSTRIA E COMÉRCIO LTDA	DIAGNÓSTICA INDÚSTRIA E COMÉRCIO LTDA	80638720081	11/27/2027

ETI-AB-HCVK-4	ANTICORPO TOTAL PARA VÍRUS DE HEPATITE C (ANTI-HCV)	DIASORIN LTDA	DIASORIN SPA.	10339840205	8/22/2015
Murex HCV Ag/Ab Combination	ANTÍGENO E ANTICORPO PARA VÍRUS DE HEPATITE C (HCV AG/ANTI-HCV)	DIASORIN LTDA	DIASORIN SPA.	10339840324	8/1/2026
Murex HCV Versão 4.0	ANTICORPO TOTAL PARA VÍRUS DE HEPATITE C (ANTI-HCV)	DIASORIN LTDA	DIASORIN SOUTH AFRICA (PTY) LTD. / DIASORIN S.P.A	10339840326	8/1/2026
LIAISON XL MUREX Control HCV Ab	ANTICORPO TOTAL PARA VÍRUS DE HEPATITE C (ANTI-HCV)	DIASORIN LTDA	DIASORIN SPA.	10339840373	7/26/2021
LIAISON XL MUREX HCV Ab	ANTICORPO TOTAL PARA VÍRUS DE HEPATITE C (ANTI-HCV)	DIASORIN LTDA	DIASORIN SPA.	10339840374	7/26/2021
Enzygnost Anti-HCV 4.0	ANTICORPO TOTAL PARA VÍRUS DE HEPATITE C (ANTI-HCV)	DIASORIN LTDA	SIEMENS HEALTHCARE DIAGNOSTICS PRODUCTS GMBH	10339840444	7/26/2021
LIAISON XL MUREX HCV Ab	ANTICORPO TOTAL PARA VÍRUS DE HEPATITE C (ANTI-HCV)	DIASORIN LTDA	DIASORIN SPA.	10339840482	4/30/2028
HCV	ANTICORPO TOTAL PARA VÍRUS DE HEPATITE C (ANTI-HCV)	DOLES REAGENTES E EQUIPAMENTOS PARA LABORATORIOS LTDA	DOLES REAGENTES E EQUIPAMENTOS PARA LABORATORIOS LTDA	10231810108	5/31/2015
HCV	ANTÍGENO DE VÍRUS DE HEPATITE C (HCV)	EBRAM PRODUTOS LABORATORIAIS LTDA	EBRAM PRODUTOS LABORATORIAIS LTDA	10159820212	9/16/2019
HCV	ANTÍGENO DE VÍRUS DE HEPATITE C (HCV)	EBRAM PRODUTOS LABORATORIAIS LTDA	HANGZHOU ALL TEST BIOTECH CO.,LTD	10159820230	11/25/2029
STANDARD Q HCV Ab Test	IMUNOGLOBULINA G PARA VÍRUS DE HEPATITE C (ANTI-HCV IGG)	EMERGO BRAZIL IMPORT IMPORTACAO E DISTRIBUICAO DE PRODUTOS MEDICOS HOSPITALARES LTDA	SD BIOSENSOR, INC.	80117580779	6/3/2029
HCV Ab Eco Teste	ANTICORPO TOTAL PARA VÍRUS DE HEPATITE C (ANTI-HCV)	Eco Diagnostica Ltda	Eco Diagnostica Ltda	80954880014	9/12/2026
ECO F HCV Ab	ANTICORPO TOTAL PARA VÍRUS DE HEPATITE C (ANTI-HCV)	Eco Diagnostica Ltda	Eco Diagnostica Ltda	80954880094	10/15/2028
INNO-LIA HCV SCORE	ANTICORPO CONFIRMATÓRIO PARA VÍRUS DE HEPATITE C (ANTI-HCV)	FUJIREBIO DIAGNÓSTICOS DO BRASIL LTDA.	INNOGENETICS N. V.	80433150006	8/31/2014

INNO-LIA™ HCV Score <20T,CE> (80538)	ANTICORPO CONFIRMATÓRIO PARA VÍRUS DE HEPATITE C (ANTI-HCV)	FUJIREBIO DIAGNÓSTICOS DO BRASIL LTDA.	FUJIREBIO EUROPE N.V.	80433150025	11/1/2020
Teste Rápido HCV Bahiafarma	ANTICORPO TOTAL PARA VÍRUS DE HEPATITE C (ANTI-HCV)	FUNDAÇÃO BAIANA DE PESQ. CIENTIFICA E DESENV. TECNOLOGICO, FORNECIMENTO E DISTRIBUIÇÃO DE MEDICAMENTOS-BAHIAFARMA	FUNDAÇÃO BAIANA DE PESQ. CIENTIFICA E DESENV. TECNOLOGICO, FORNECIMENTO E DISTRIBUIÇÃO DE MEDICAMENTOS-BAHIAFARMA	81285200011	10/16/2027
HCV - EIC	ANTICORPO TOTAL PARA VÍRUS DE HEPATITE C (ANTI-HCV)	GOLD ANALISA DIAGNÓSTICA LTDA	GOLD ANALISA DIAGNÓSTICA LTDA	80022230165	6/15/2024
HCV Ab	ANTICORPO TOTAL PARA VÍRUS DE HEPATITE C (ANTI-HCV)	GOYAZES BIOTECNOLOGIA LTDA	DIA PRO DIAGNOSTIC BIOPROBES S.R.L.	80345000013	1/7/2028
GB-NANBASE C-96 4.0	ANTICORPO TOTAL PARA VÍRUS DE HEPATITE C (ANTI-HCV)	GOYAZES BIOTECNOLOGIA LTDA	GENERAL BIOLOGICALS CORP.	80345000224	11/28/2016
ANTI - HCV	Detec. ou Quantific. Antig. Anticorpos Hepatite C	IN VITRO DIAGNOSTICA LTDA	HUMAN GMBH	10303460222	10/1/2012
ANTI-HCV ELISA	ANTICORPO TOTAL PARA VÍRUS DE HEPATITE C (ANTI-HCV)	IN VITRO DIAGNOSTICA LTDA	IN VITRO DIAGNOSTICA LTDA	10303460443	5/14/2017
Hexagon HCV	ANTICORPO TOTAL PARA VÍRUS DE HEPATITE C (ANTI-HCV)	IN VITRO DIAGNOSTICA LTDA	IN VITRO DIAGNOSTICA LTDA	10303460471	3/5/2028
HCV	ANTICORPO TOTAL PARA VÍRUS DE HEPATITE C (ANTI-HCV)	INTERTECK INTERNACIONAL IMPORTAÇÃO E EXPORTAÇÃO LTDA.	ACON BIOTECH (HANGZHOU) CO., LTD	10402200082	2/1/2025
TOYO HCV CASSETTE TEST KIT	ANTICORPO TOTAL PARA VÍRUS DE HEPATITE C (ANTI-HCV)	JC Pharma & Health Comércio, Exportação e Importação Ltda	TURKLAB TIBBI MALZEMELER SAN. TIC. A.S.	80721060034	11/3/2025
KIT DE REAGENTE IMUNODIAGNOSTICO VITROS* PARA ANTI HCV	ANTICORPO TOTAL PARA VÍRUS DE HEPATITE C (ANTI-HCV)	JOHNSON & JOHNSON DO BRASIL INDÚSTRIA E COMÉRCIO DE PRODUTOS PARA SAÚDE LTDA	ORTHO-CLINICAL DIAGNOSTICS	10132590629	11/26/2017
CALIBRADOR IMUNODIAGNOSTICO VITROS* PARA ANTI HCV	ANTICORPO TOTAL PARA VÍRUS DE HEPATITE C (ANTI-HCV)	JOHNSON & JOHNSON DO BRASIL INDÚSTRIA E COMÉRCIO DE PRODUTOS PARA SAÚDE LTDA	ORTHO-CLINICAL DIAGNOSTICS	10132590633	11/26/2017

CONTROLE IMUNODIAGNOSTICO VITROS* PARA ANTI-HCV	ANTICORPO TOTAL PARA VÍRUS DE HEPATITE C (ANTI-HCV)	JOHNSON & JOHNSON DO BRASIL INDÚSTRIA E COMÉRCIO DE PRODUTOS PARA SAÚDE LTDA	ORTHO-CLINICAL DIAGNOSTICS	10132590639	11/26/2017
ORTHO HCV 3.0 ELISA Test System with Enhanced SAVe (Sample Addition Verification)	ANTICORPO TOTAL PARA VÍRUS DE HEPATITE C (ANTI-HCV)	JOHNSON & JOHNSON DO BRASIL INDÚSTRIA E COMÉRCIO DE PRODUTOS PARA SAÚDE LTDA	ORTHO-CLINICAL DIAGNOSTICS	80145901810	11/26/2017
Teste Rápido – HCV Fluxo Reverso	ANTÍGENO DE VÍRUS DE HEPATITE C (HCV)	KATAL BIOTECNOLOGICA INDÚSTRIA E COMÉRCIO LTDA	KATAL BIOTECNOLOGICA INDÚSTRIA E COMÉRCIO LTDA	10377390178	4/29/2019
HCV	ANTICORPO TOTAL PARA VÍRUS DE HEPATITE C (ANTI-HCV)	KATAL BIOTECNOLOGICA INDÚSTRIA E COMÉRCIO LTDA	KATAL BIOTECNOLOGICA INDÚSTRIA E COMÉRCIO LTDA	10377390212	10/5/2025
HCV	ANTICORPO TOTAL PARA VÍRUS DE HEPATITE C (ANTI-HCV)	KATAL BIOTECNOLOGICA INDÚSTRIA E COMÉRCIO LTDA	KATAL BIOTECNOLOGICA INDÚSTRIA E COMÉRCIO LTDA	10377390242	12/16/2029
ANTI HCV SYM SOLUTION	ANTICORPO TOTAL PARA VÍRUS DE HEPATITE C (ANTI-HCV)	KHAYROS DIAGNOSTICA FABRICACAO, COMERCIALIZACAO E DISTRIBUICAO DE PRODUTOS LTDA	KHAYROS DIAGNOSTICA FABRICACAO, COMERCIALIZACAO E DISTRIBUICAO DE PRODUTOS LTDA	80105220043	12/22/2019
Q-PREVEN Anti HCV-DBS	ANTICORPO TOTAL PARA VÍRUS DE HEPATITE C (ANTI-HCV)	KHAYROS DIAGNOSTICA FABRICACAO, COMERCIALIZACAO E DISTRIBUICAO DE PRODUTOS LTDA	KHAYROS DIAGNOSTICA FABRICACAO, COMERCIALIZACAO E DISTRIBUICAO DE PRODUTOS LTDA	80105220078	12/22/2019
Smart Test Anti HCV SYM	ANTICORPO TOTAL PARA VÍRUS DE HEPATITE C (ANTI-HCV)	KHAYROS DIAGNOSTICA FABRICACAO, COMERCIALIZACAO E DISTRIBUICAO DE PRODUTOS LTDA	KHAYROS DIAGNOSTICA FABRICACAO, COMERCIALIZACAO E DISTRIBUICAO DE PRODUTOS LTDA	80105220107	12/22/2019
Q-PREVEN Anti HCV-DBS	ANTICORPO TOTAL PARA VÍRUS DE HEPATITE C (ANTI-HCV)	KHAYROS DIAGNOSTICA FABRICACAO, COMERCIALIZACAO E DISTRIBUICAO DE PRODUTOS LTDA	KHAYROS DIAGNOSTICA FABRICACAO, COMERCIALIZACAO E DISTRIBUICAO DE PRODUTOS LTDA	80105220109	9/23/2025
HCV ELISA 3ª Generación	Detec. ou Quantific. Antig. Anticorpos Hepatite C	LABINBRAZ COMERCIAL LTDA	WIENER LABORATORIOS S.A.I.C	10268590245	9/24/2024
Família Antibody to Hepatitis C Virus (Anti-HCV)	ANTICORPO TOTAL PARA VÍRUS DE HEPATITE C (ANTI-HCV)	LABORLAB PRODUTOS PARA LABORATÓRIOS LTDA EPP	WIENER LABORATÓRIO S.A.I.C.	10246810307	12/3/2028
HCV	Detec. ou Quantific. Antig. Anticorpos Hepatite C	LABTEST DIAGNOSTICA S/A	LABTEST DIAGNOSTICA S/A	10009010147	10/1/2012
anti-HCV	ANTICORPO TOTAL PARA VÍRUS DE HEPATITE C (ANTI-HCV)	LABTEST DIAGNOSTICA S/A	LABTEST DIAGNOSTICA S/A	10009010269	4/1/2023

Lab Rapid HCV	ANTICORPO TOTAL PARA VÍRUS DE HEPATITE C (ANTI-HCV)	LABTEST DIAGNOSTICA S/A	LABTEST DIAGNOSTICA S/A	10009010359	8/10/2030
TESTE RAPIDO DE ANTICORPO ORAQUICK HCV	ANTICORPO TOTAL PARA VÍRUS DE HEPATITE C (ANTI-HCV)	LD COMERCIO DE MATERIAIS PARA DIAGNOSTICO E MEDICO HOSPITALAR LTDA	ORASURE TECHNOLOGIES, INC.	80686840001	6/2/2024
LUMIRATEK HCV - CASSETE	ANTICORPO TOTAL PARA VÍRUS DE HEPATITE C (ANTI-HCV)	LUMIRADX HEALTHCARE LTDA	HANGZHOU BIOTEST BIOTECH CO., LTD	81327670083	7/31/2027
Imunoscreen HCV SS	ANTÍGENO DE VÍRUS DE HEPATITE C (HCV)	MBIOLOG DIAGNOSTICOS LTDA	MBIOLOG DIAGNOSTICOS LTDA	80047580150	1/10/2016
IMUNOSCREEN HCV SS	ANTICORPO TOTAL PARA VÍRUS DE HEPATITE C (ANTI-HCV)	MBIOLOG DIAGNOSTICOS LTDA	MBIOLOG DIAGNOSTICOS LTDA	80047580182	9/5/2026
IMUNOCROM MB HCV	ANTICORPO TOTAL PARA VÍRUS DE HEPATITE C (ANTI-HCV)	MBIOLOG DIAGNOSTICOS LTDA	MBIOLOG DIAGNOSTICOS LTDA	80047580186	2/6/2027
MedTeste HCV	ANTICORPO TOTAL PARA VÍRUS DE HEPATITE C (ANTI-HCV)	MEDLEVENSOHN COMÉRCIO E REPRESENTAÇÕES DE PRODUTOS HOSPITALARES LTDA	HANGZHOU BIOTEST BIOTECH CO. LTD.	80560310019	4/3/2027
FAMÍLIA MEDTESTE HCV TESTE RAPIDO	ANTICORPO TOTAL PARA VÍRUS DE HEPATITE C (ANTI-HCV)	MEDLEVENSOHN COMÉRCIO E REPRESENTAÇÕES DE PRODUTOS HOSPITALARES LTDA	HANGZHOU BIOTEST BIOTECH CO., LTD	80560310051	1/27/2030
FAMÍLIA Anti-HCV (CLIA)	ANTICORPO TOTAL PARA VÍRUS DE HEPATITE C (ANTI-HCV)	MINDRAY DO BRASIL COMÉRCIO E DISTRIBUIÇÃO DE EQUIPAMENTOS MÉDICOS LTDA.	SHENZHEN MINDRAY BIO-MEDICAL ELETRONICS CO. LTDA	80943610089	8/13/2028
HCV BLOT 3.0 WESTERN BLOT ASSAY	ANTICORPO CONFIRMATÓRIO PARA VÍRUS DE HEPATITE C (ANTI-HCV)	MP BIOMEDICALS DO BRASIL LTDA	MP BIOMEDICALS ASIA PACIFIC PTE LTD	80313210022	2/22/2026
ORTHO HCV 3.0 ELISA Test System with Enhanced SAVe (Sample Addition Verification)	ANTICORPO TOTAL PARA VÍRUS DE HEPATITE C (ANTI-HCV)	ORTHO CLINICAL DIAGNÓSTICS DO BRASIL PRODUTOS PARA SAÚDE LTDA	ORTHO-CLINICAL DIAGNOSTICS	81246980014	2/22/2021
CONTROLE IMUNODIAGNOSTICO VITROS* PARA ANTI-HCV	ANTICORPO TOTAL PARA VÍRUS DE HEPATITE C (ANTI-HCV)	ORTHO CLINICAL DIAGNÓSTICS DO BRASIL PRODUTOS PARA SAÚDE LTDA	ORTHO-CLINICAL DIAGNOSTICS	81246986796	10/23/2026

CALIBRADOR IMUNODIAGNOSTICO VITROS* PARA ANTI HCV	ANTICORPO TOTAL PARA VÍRUS DE HEPATITE C (ANTI-HCV)	ORTHO CLINICAL DIAGNÓSTICS DO BRASIL PRODUTOS PARA SAÚDE LTDA	ORTHO-CLINICAL DIAGNOSTICS	81246986814	10/23/2026
KIT DE REAGENTE IMUNODIAGNOSTICO VITROS* PARA ANTI HCV	ANTICORPO TOTAL PARA VÍRUS DE HEPATITE C (ANTI-HCV)	ORTHO CLINICAL DIAGNÓSTICS DO BRASIL PRODUTOS PARA SAÚDE LTDA	ORTHO-CLINICAL DIAGNOSTICS	81246986817	10/23/2026
LG HCD 3.0 PLUS	ANTICORPO TOTAL PARA VÍRUS DE HEPATITE C (ANTI-HCV)	PLAST LABOR IND E COM DE EQUIP HOSP E LABORATORIO LTDA	LG LIFE SCIENCES	80035670015	11/17/2013
IMMUNOCOMB II HCV	ANTICORPO TOTAL PARA VÍRUS DE HEPATITE C (ANTI-HCV)	PRODIMOL BIOTECNOLOGIA S/A	ORGENICS LTD	80195040074	5/13/2013
Família Teste rápido em Cassete de HCV	ANTICORPO TOTAL PARA VÍRUS DE HEPATITE C (ANTI-HCV)	QR Consulting, Importação e Distribuição de Produtos Médicos Ltda	ACRO BIOTECH INC	81325990114	3/16/2030
HCV Hepatitis C Vírus Rapid Test Device (Sangue Total/Soro/Plasma)	ANTICORPO TOTAL PARA VÍRUS DE HEPATITE C (ANTI-HCV)	QUANTUM DIAGNÓSTICOS LTDA	ABON BIOPHARM CO, LTD.	80242750148	3/30/2015
HCV TEST BIOEASY	ANTICORPO TOTAL PARA VÍRUS DE HEPATITE C (ANTI-HCV)	QUANTUM DIAGNÓSTICOS LTDA	ABON BIOPHARM CO, LTD.	80242750182	4/23/2017
BIOLISA HCV	ANTICORPO TOTAL PARA VÍRUS DE HEPATITE C (ANTI-HCV)	QUIBASA QUÍMICA BÁSICA LTDA	QUIBASA QUÍMICA BÁSICA LTDA	10269360205	1/10/2016
HCV	ANTICORPO TOTAL PARA VÍRUS DE HEPATITE C (ANTI-HCV)	QUIBASA QUÍMICA BÁSICA LTDA	QUIBASA QUÍMICA BÁSICA LTDA	10269360231	3/12/2027
BIOLISA HCV	ANTICORPO TOTAL PARA VÍRUS DE HEPATITE C (ANTI-HCV)	QUIBASA QUÍMICA BÁSICA LTDA	QUIBASA QUÍMICA BÁSICA LTDA	10269360304	4/25/2026
HCV Ab	Detec.ou Quantific.Antig.Anticorpos Hepatite C	RADIM LATINO AMERICA DIAGNOSTICO LTDA.	RADIM SPA	80103990078	7/2/2012
ORTHO HCV 3.0 ELISA Test System Enhanced SAVe	ANTICORPO TOTAL PARA VÍRUS DE HEPATITE C (ANTI-HCV)	REM INDUSTRIA E COMERCIO LTDA	ORTHO CLINICAL DIAGNOSTICS	10269410002	4/23/2016
CHIRON RIBA HCV 3.0 SIA	ANTICORPO CONFIRMATÓRIO PARA VÍRUS DE HEPATITE C (ANTI-HCV)	REM INDUSTRIA E COMERCIO LTDA	NOVARTIS VACCINES AND DIAGNOSTICS, INC.	10269410395	8/23/2014

DETECT PARA HCV	Detec.ou Quantific.Antig.Anticorpos Hepatite C	RESSERV COMÉRCIO DE PRODUTOS DIAGNOSTICOS LTDA ME	ADALTIS INC	80213250181	1/2/2012
EIAGEN HCV Ab (v.4) KIT	ANTICORPO TOTAL PARA VÍRUS DE HEPATITE C (ANTI-HCV)	RESSERV COMÉRCIO DE PRODUTOS DIAGNOSTICOS LTDA ME	ADALTIS S.R.L.	80213250217	2/11/2013
recomLine HCV IgG	ANTICORPO CONFIRMATÓRIO PARA VÍRUS DE HEPATITE C (ANTI-HCV)	RESSERV COMÉRCIO DE PRODUTOS DIAGNOSTICOS LTDA ME	MIKROGEN GMBH	80213250236	8/18/2019
ANTI-HCV ELISA KIT	ANTICORPO TOTAL PARA VÍRUS DE HEPATITE C (ANTI-HCV)	RESSERV COMÉRCIO DE PRODUTOS DIAGNOSTICOS LTDA ME	AUTOBIO DIAGNOSTICS CO., LTD	80213250394	1/11/2025
HCVAb	ANTICORPO TOTAL PARA VÍRUS DE HEPATITE C (ANTI-HCV)	RESSERV COMÉRCIO DE PRODUTOS DIAGNOSTICOS LTDA ME	M.B.S. S.R.L.	80213250454	9/21/2015
ELECSYS ANTI- HCV	ANTÍGENO DE VÍRUS DE HEPATITE C (HCV)	ROCHE DIAGNÓSTICA BRASIL LTDA	ROCHE DIAGNOSTICS GMBH	10287410760	9/22/2013
PRECICONTROL ANTI- HCV	ANTICORPO TOTAL PARA VÍRUS DE HEPATITE C (ANTI-HCV)	ROCHE DIAGNÓSTICA BRASIL LTDA	ROCHE DIAGNOSTICS GMBH (MANNHEIM)	10287410764	10/27/2023
Elecsys Anti-HCV II	ANTICORPO TOTAL PARA VÍRUS DE HEPATITE C (ANTI-HCV)	ROCHE DIAGNÓSTICA BRASIL LTDA	ROCHE DIAGNOSTICS GMBH	10287410980	10/29/2027
Elecsys Anti-HCV II	ANTICORPO TOTAL PARA VÍRUS DE HEPATITE C (ANTI-HCV)	ROCHE DIAGNÓSTICA BRASIL LTDA	ROCHE DIAGNOSTICS GMBH	10287411317	3/26/2028
SD HCV ELISA 3.0	ANTICORPO TOTAL PARA VÍRUS DE HEPATITE C (ANTI-HCV)	RZ DE OLIVEIRA DIAGNÓSTICA EPP	STANDARD DIAGNOSTICS INC.	80313040031	6/23/2013
SB BIOLINE HCV	ANTÍGENO DE VÍRUS DE HEPATITE C (HCV)	RZ DE OLIVEIRA DIAGNÓSTICA EPP	STANDARD DIAGNOSTICS INC.	80313040034	6/30/2013
HCV Ab	ANTICORPO TOTAL PARA VÍRUS DE HEPATITE C (ANTI-HCV)	SERION BRASIL IMPORTAÇÃO E DISTRIBUIÇÃO DE PRODUTOS DIAGNÓSTICOS LTDA	DIA.PRO DIAGNOSTIC BIOPROBES SRL	80826840137	12/16/2029
QC HCV CENTAUR	ANTICORPO TOTAL PARA VÍRUS DE HEPATITE C (ANTI-HCV)	SIEMENS HEALTHCARE DIAGNÓSTICOS LTDA	SIEMENS HEALTHCARE DIAGNIOSTICS INC.	10345160591	7/9/2027
ADVIA CENTAUR HCV	ANTICORPO TOTAL PARA VÍRUS DE HEPATITE C (ANTI-HCV)	SIEMENS HEALTHCARE DIAGNÓSTICOS LTDA	SIEMENS HEALTHCARE DIAGNOSITCS INC	10345160632	8/27/2027

VERSANT AUTO DETECT SET	ANTÍGENO DE VÍRUS DE HEPATITE C (HCV)	SIEMENS HEALTHCARE DIAGNÓSTICOS LTDA	SIEMENS HEALTHCARE DIAGNOSITCS INC	10345160843	8/11/2013
SERODIA HCV	ANTICORPO TOTAL PARA VÍRUS DE HEPATITE C (ANTI-HCV)	SIEMENS HEALTHCARE DIAGNÓSTICOS LTDA	FUJIREBIO INC.	10345161665	12/28/2014
Enzygnost Anti-HCV 4.0	ANTICORPO TOTAL PARA VÍRUS DE HEPATITE C (ANTI-HCV)	SIEMENS HEALTHCARE DIAGNÓSTICOS LTDA	SIEMENS HEALTHCARE DIAGNOSTICS PRODUCTS GMBH	10345161888	22/04/2018
Família Atellica IM Hepatite C (aHCV)	IMUNOGLOBULINA G PARA VÍRUS DE HEPATITE C (ANTI-HCV IGG)	SIEMENS HEALTHCARE DIAGNÓSTICOS LTDA	SIEMENS HEALTHCARE DIAGNOSITCS INC	10345162300	10/15/2028
HCV BLOT 3.0 WESTERN BLOT ASSAY	ANTICORPO CONFIRMATÓRIO PARA VÍRUS DE HEPATITE C (ANTI-HCV)	VR MEDICAL IMPORTADORA E DISTRIBUIDORA DE PRODUTOS MÉDICOS LTDA	MP BIOMEDICALS ASIA PACIFIC PTE LTD	80102510928	9/1/2020
FAMÍLIA Anti-HCV (CLIA)	ANTICORPO TOTAL PARA VÍRUS DE HEPATITE C (ANTI-HCV)	VR MEDICAL IMPORTADORA E DISTRIBUIDORA DE PRODUTOS MÉDICOS LTDA	SHENZHEN MINDRAY BIO-MEDICAL ELETRONICS CO. LTDA.	80102512094	9/1/2020
MAGLUMI™ Anti-HCV (CLIA)	ANTICORPO TOTAL PARA VÍRUS DE HEPATITE C (ANTI-HCV)	VR MEDICAL IMPORTADORA E DISTRIBUIDORA DE PRODUTOS MÉDICOS LTDA	SHENZHEN MINDRAY BIO-MEDICAL ELETRONICS CO. LTDA.	80102512285	7/29/2029
INNO-LIA™ HCV Score <20T,CE> (80538)	ANTICORPO TOTAL PARA VÍRUS DE HEPATITE C (ANTI-HCV)	VR MEDICAL IMPORTADORA E DISTRIBUIDORA DE PRODUTOS MÉDICOS LTDA	FUJIREBIO EUROPE N.V.	80102512528	3/23/2025
Q-PREVEN Anti HCV-DBS	ANTICORPO TOTAL PARA VÍRUS DE HEPATITE C (ANTI-HCV)	VYTTRA DIAGNOSTICOS IMPORTACAO E EXPORTACAO S.A.	VYTTRA DIAGNOSTICOS IMPORTACAO E EXPORTACAO S.A.	81692610117	5/10/2020
Smart Test Anti HCV SYM	ANTICORPO TOTAL PARA VÍRUS DE HEPATITE C (ANTI-HCV)	VYTTRA DIAGNOSTICOS IMPORTACAO E EXPORTACAO S.A.	VYTTRA DIAGNOSTICOS IMPORTACAO E EXPORTACAO S.A.	81692610120	3/5/2028
ANTI HCV SYM SOLUTION	ANTICORPO TOTAL PARA VÍRUS DE HEPATITE C (ANTI-HCV)	VYTTRA DIAGNOSTICOS IMPORTACAO E EXPORTACAO S.A.	VYTTRA DIAGNOSTICOS IMPORTACAO E EXPORTACAO S.A.	81692610145	6/26/2026
recomLine HCV IgG	ANTICORPO CONFIRMATÓRIO PARA VÍRUS DE HEPATITE C (ANTI-HCV)	VYTTRA DIAGNOSTICOS IMPORTACAO E EXPORTACAO S.A.	MIKROGEN GMBH	10300390664	5/19/2023
HCV RÁPIDO	ANTICORPO TOTAL PARA VÍRUS DE HEPATITE C (ANTI-HCV)	Vida Biotecnologia Ltda - ME	Vida Biotecnologia Ltda - ME	80785070066	7/28/2024

IMUNO-RÁPIDO HCV	ANTICORPO TOTAL PARA VÍRUS DE HEPATITE C (ANTI-HCV)	WAMA PRODUTOS PARA LABORATORIO LTDA	WAMA PRODUTOS PARA LABORATORIO LTDA	10310030092	1/2/2027
Imuno-ELISA anti-HCV	ANTICORPO TOTAL PARA VÍRUS DE HEPATITE C (ANTI-HCV)	WAMA PRODUTOS PARA LABORATORIO LTDA	WAMA PRODUTOS PARA LABORATORIO LTDA	10310030130	11/28/2016
BioPix HCV	ANTICORPO TOTAL PARA VÍRUS DE HEPATITE C (ANTI-HCV)	WAMA PRODUTOS PARA LABORATORIO LTDA	WAMA PRODUTOS PARA LABORATORIO LTDA	10310030133	9/5/2026
BIOELISA HCV 4.0	ANTICORPO TOTAL PARA VÍRUS DE HEPATITE C (ANTI-HCV)	WERFEN MEDICAL LTDA	BIOKIT S/A	80003610141	5/31/2021
Bioblot HCV - 18 TESTES	ANTICORPO CONFIRMATÓRIO PARA VÍRUS DE HEPATITE C (ANTI-HCV)	WERFEN MEDICAL LTDA	BIOKIT S/A	80003610256	31/05/2021
BIO-FLASH anti-HCV Calibradores	ANTICORPO TOTAL PARA VÍRUS DE HEPATITE C (ANTI-HCV)	WERFEN MEDICAL LTDA	BIOKIT S/A	80003610337	31/05/2021
BIO-FLASH anti-HCV Controles	ANTICORPO TOTAL PARA VÍRUS DE HEPATITE C (ANTI-HCV)	WERFEN MEDICAL LTDA	BIOKIT S/A	80003610338	5/31/2021
BIO-FLASH anti-HCV	ANTICORPO TOTAL PARA VÍRUS DE HEPATITE C (ANTI-HCV)	WERFEN MEDICAL LTDA	BIOKIT S/A	80003610358	5/31/2021

Source: https://consultas.anvisa.gov.br/#/saude/25351321690201704/?nomeTecnico=hepatite%20c, as of September 1st 2021. Double-checked after ANVISA final response to or official request – Protocol 2021195961 (September 3, 2021).
