# Supplementary material for: Persistently high hepatitis C rates in haemodialysis patients in Brazil [a systematic review and meta-analysis]
Source: Sci Rep. 2022 Jan 10;12:330. doi: 10.1038/s41598-021-03961-x (PMC8748660; doi:10.1038/s41598-021-03961-x)
Supplement: Supplementary file 6 — Supplementary Information 6. [file 41598_2021_3961_MOESM6_ESM.pdf]

Table 6S: PRISMA 2020 Checklist

Section and Topic	Item #	Checklist item	Location where item is reported
**TITLE**			
Title	1	Identify the report as a systematic review.	Line 1
**ABSTRACT**			
Abstract	2	See the PRISMA 2020 for Abstracts checklist.	Abstract
**INTRODUCTION**			
Rationale	3	Describe the rationale for the review in the context of existing knowledge.	Lines 92-99
Objectives	4	Provide an explicit statement of the objective(s) or question(s) the review addresses.	Lines 127-128
**METHODS**			
Eligibility criteria	5	Specify the inclusion and exclusion criteria for the review and how studies were grouped for the syntheses.	Lines 166-204
Information sources	6	Specify all databases, registers, websites, organisations, reference lists and other sources searched or consulted to identify studies. Specify the date when each source was last searched or consulted.	Lines 144-146
Search strategy	7	Present the full search strategies for all databases, registers and websites, including any filters and limits used.	Lines 145-146; Revised Web appendix 1. Figure 1
Selection process	8	Specify the methods used to decide whether a study met the inclusion criteria of the review, including how many reviewers screened each record and each report retrieved, whether they worked independently, and if applicable, details of automation tools used in the process.	Lines 212-219
Data collection process	9	Specify the methods used to collect data from reports, including how many reviewers collected data from each report, whether they worked independently, any processes for obtaining or confirming data from study investigators, and if applicable, details of automation tools used in the process.	Lines 220-231
Data items	10a	List and define all outcomes for which data were sought. Specify whether all results that were compatible with each outcome domain in each study were sought (e.g. for all measures, time points, analyses), and if not, the methods used to decide which results to collect.	Lines 234-237;243-246

Section and Topic	Item #	Checklist item	Location where item is reported
	10b	List and define all other variables for which data were sought (e.g. participant and intervention characteristics, funding sources). Describe any assumptions made about any missing or unclear information.	Lines 220-228
Study risk of bias assessment	11	Specify the methods used to assess risk of bias in the included studies, including details of the tool(s) used, how many reviewers assessed each study and whether they worked independently, and if applicable, details of automation tools used in the process.	Lines 261-264
Effect measures	12	Specify for each outcome the effect measure(s) (e.g. risk ratio, mean difference) used in the synthesis or presentation of results.	Lines 250-256
Synthesis methods	13a	Describe the processes used to decide which studies were eligible for each synthesis (e.g. tabulating the study intervention characteristics and comparing against the planned groups for each synthesis (item #5)).	Lines 213-221
	13b	Describe any methods required to prepare the data for presentation or synthesis, such as handling of missing summary statistics, or data conversions.	Lines 220-221
	13c	Describe any methods used to tabulate or visually display results of individual studies and syntheses.	Lines 247-260
	13d	Describe any methods used to synthesize results and provide a rationale for the choice(s). If meta-analysis was performed, describe the model(s), method(s) to identify the presence and extent of statistical heterogeneity, and software package(s) used.	Lines 261-273
	13e	Describe any methods used to explore possible causes of heterogeneity among study results (e.g. subgroup analysis, meta-regression).	Lines 266-269
	13f	Describe any sensitivity analyses conducted to assess robustness of the synthesized results.	Lines 261-264
Reporting bias assessment	14	Describe any methods used to assess risk of bias due to missing results in a synthesis (arising from reporting biases).	Web appendix 3<br>Web appendix 4<br>Main body of the text (Methods, Results and Discussion)
Certainty assessment	15	Describe any methods used to assess certainty (or confidence) in the body of evidence for an outcome.	Lines 257-258

Section and Topic	Item #	Checklist item	Location where item is reported
**RESULTS**			
Study selection	16a	Describe the results of the search and selection process, from the number of records identified in the search to the number of studies included in the review, ideally using a flow diagram.	Lines 291-305
	16b	Cite studies that might appear to meet the inclusion criteria, but which were excluded, and explain why they were excluded.	Lines 303-305
Study characteristics	17	Cite each included study and present its characteristics.	Lines 306-316
Risk of bias in studies	18	Present assessments of risk of bias for each included study.	Line 415
Results of individual studies	19	For all outcomes, present, for each study: (a) summary statistics for each group (where appropriate) and (b) an effect estimate and its precision (e.g. confidence/credible interval), ideally using structured tables or plots.	Lines 348-364
Results of syntheses	20a	For each synthesis, briefly summarise the characteristics and risk of bias among contributing studies.	Web appendix 3
	20b	Present results of all statistical syntheses conducted. If meta-analysis was done, present for each the summary estimate and its precision (e.g. confidence/credible interval) and measures of statistical heterogeneity. If comparing groups, describe the direction of the effect.	Lines 358-361;382-392
	20c	Present results of all investigations of possible causes of heterogeneity among study results.	Lines 362-378
	20d	Present results of all sensitivity analyses conducted to assess the robustness of the synthesized results.	Lines 362-378
Reporting biases	21	Present assessments of risk of bias due to missing results (arising from reporting biases) for each synthesis assessed.	Lines 362-378
Certainty of evidence	22	Present assessments of certainty (or confidence) in the body of evidence for each outcome assessed.	Lines 308-309;330
**DISCUSSION**			
Discussion	23a	Provide a general interpretation of the results in the context of other evidence.	Lines 450-451
	23b	Discuss any limitations of the evidence included in the review.	Lines 463-494
	23c	Discuss any limitations of the review processes used.	Lines 463-494
	23d	Discuss implications of the results for practice, policy, and future research.	Lines 537-543

Section and Topic	Item #	Checklist item	Location where item is reported
**OTHER INFORMATION**			
Registration and protocol	24a	Provide registration information for the review, including register name and registration number, or state that the review was not registered.	Line 54
	24b	Indicate where the review protocol can be accessed, or state that a protocol was not prepared.	Registration available at PROSPERO
	24c	Describe and explain any amendments to information provided at registration or in the protocol.	None
Support	25	Describe sources of financial or non-financial support for the review, and the role of the funders or sponsors in the review.	Lines 29-32
Competing interests	26	Declare any competing interests of review authors.	Lines 40-41
Availability of data, code and other materials	27	Report which of the following are publicly available and where they can be found: template data collection forms; data extracted from included studies; data used for all analyses; analytic code; any other materials used in the review.	Revised Web appendix 1; Web appendix 2, Web appendix 3

PRIMSA Abstract Checklist

Section and Topic	Item #	Checklist item	Reported (Yes/No)
**TITLE**			
Title	1	Identify the report as a systematic review.	Yes, depending on the editors final decision. Defined as such in the Cover letter and Revised text with track changes
**BACKGROUND**			
Objectives	2	Provide an explicit statement of the main objective(s) or question(s) the review addresses.	Yes
**METHODS**			
Eligibility criteria	3	Specify the inclusion and exclusion criteria for the review.	Yes
Information sources	4	Specify the information sources (e.g. databases, registers) used to identify studies and the date when each was last searched.	Yes
Risk of bias	5	Specify the methods used to assess risk of bias in the included studies.	Yes
Synthesis of results	6	Specify the methods used to present and synthesise results.	Yes
**RESULTS**			
Included studies	7	Give the total number of included studies and participants and summarise relevant characteristics of studies.	Yes
Synthesis of results	8	Present results for main outcomes, preferably indicating the number of included studies and participants for each. If meta-analysis was done, report the summary estimate and confidence/credible interval. If comparing groups, indicate the direction of the effect (i.e. which group is favoured).	Yes
**DISCUSSION**			
Limitations of	9	Provide a brief summary of the limitations of the evidence included in the review (e.g.	Yes, now a

Section and Topic	Item #	Checklist item	Reported (Yes/No)
evidence		study risk of bias, inconsistency and imprecision).	specific section of the revised Discussion highlighted in bold
Interpretation	10	Provide a general interpretation of the results and important implications.	Yes
**OTHER**			
Funding	11	Specify the primary source of funding for the review.	Yes, stated in the beginning of the text
Registration	12	Provide the register name and registration number.	Yes

Reference:

Page, M.J., *et al*. The PRISMA 2020 statement: an updated guideline for reporting systematic reviews. *BMJ* 372 (2021)
